# Lighting the way: Compelling open questions in photosynthesis research

**DOI:** 10.1093/plcell/koae203

**Published:** 2024-07-22

**Authors:** Nancy A Eckardt, Yagut Allahverdiyeva, Clarisa E Alvarez, Claudia Büchel, Adrien Burlacot, Tanai Cardona, Emma Chaloner, Benjamin D Engel, Arthur R Grossman, Dvir Harris, Nicolas Herrmann, Michael Hodges, Jan Kern, Tom Dongmin Kim, Veronica G Maurino, Conrad W Mullineaux, Henna Mustila, Lauri Nikkanen, Gabriela Schlau-Cohen, Marcos A Tronconi, Wojciech Wietrzynski, Vittal K Yachandra, Junko Yano

**Affiliations:** The Plant Cell, American Society of Plant Biologists, USA; Molecular Plant Biology Unit, Department of Life Technologies, University of Turku, 20014 Turku, Finland; Centro de Estudios Fotosintéticos y Bioquímicos (CEFOBI-CONICET), Facultad de Ciencias Bioquímicas y Farmacuticas, University of Rosario, Suipacha 570, 2000 Rosario, Argentina; Institute of Molecular Biosciences, Goethe University Frankfurt, 60438 Frankfurt, Germany; Division of Bioscience and Engineering, Carnegie Institution for Science, 260 Panama Street, Stanford, CA 94305, USA; Department of Biology, Stanford University, Stanford, CA 94305, USA; School of Biological and Behavioural Sciences, Queen Mary University of London, Mile End Road, London E1 4NS, UK; Department of Life Sciences, Imperial College London, London SW7 2AZ, UK; School of Biological and Behavioural Sciences, Queen Mary University of London, Mile End Road, London E1 4NS, UK; Department of Life Sciences, Imperial College London, London SW7 2AZ, UK; Biozentrum, University of Basel, Sptialstrasse 41, 4056 Basel, Switzerland; Division of Bioscience and Engineering, Carnegie Institution for Science, 260 Panama Street, Stanford, CA 94305, USA; Department of Biology, Stanford University, Stanford, CA 94305, USA; Department of Chemistry, Massachusetts Institute of Technology, Massachusetts Ave, Cambridge, MA 02139, USA; Institute of Molecular Biosciences, Goethe University Frankfurt, 60438 Frankfurt, Germany; Université Paris-Saclay, CNRS, INRAE, Université d’Evry, Université de Paris Cité, Institute of Plant Sciences Paris-Saclay (IPS2), 91190 Gif-sur-Yvette, France; Molecular Biophysics and Integrated Bioimaging Division, Lawrence Berkeley National Laboratory, Berkeley, CA 94720, USA; School of Biological and Behavioural Sciences, Queen Mary University of London, Mile End Road, London E1 4NS, UK; Department of Life Sciences, Imperial College London, London SW7 2AZ, UK; Molecular Plant Physiology, Institute for Cellular and Molecular Botany (IZMB), University of Bonn, Kirschallee 1, 53115 Bonn, Germany; School of Biological and Behavioural Sciences, Queen Mary University of London, Mile End Road, London E1 4NS, UK; Molecular Plant Biology Unit, Department of Life Technologies, University of Turku, 20014 Turku, Finland; Molecular Plant Biology Unit, Department of Life Technologies, University of Turku, 20014 Turku, Finland; Department of Chemistry, Massachusetts Institute of Technology, Massachusetts Ave, Cambridge, MA 02139, USA; Centro de Estudios Fotosintéticos y Bioquímicos (CEFOBI-CONICET), Facultad de Ciencias Bioquímicas y Farmacuticas, University of Rosario, Suipacha 570, 2000 Rosario, Argentina; Biozentrum, University of Basel, Sptialstrasse 41, 4056 Basel, Switzerland; Molecular Biophysics and Integrated Bioimaging Division, Lawrence Berkeley National Laboratory, Berkeley, CA 94720, USA; Molecular Biophysics and Integrated Bioimaging Division, Lawrence Berkeley National Laboratory, Berkeley, CA 94720, USA

## Abstract

Photosynthesis—the conversion of energy from sunlight into chemical energy—is essential for life on Earth. Yet there is much we do not understand about photosynthetic energy conversion on a fundamental level: how it evolved and the extent of its diversity, its dynamics, and all the components and connections involved in its regulation. In this commentary, researchers working on fundamental aspects of photosynthesis including the light-dependent reactions, photorespiration, and C_4_ photosynthetic metabolism pose and discuss what they view as the most compelling open questions in their areas of research.

## Teaser

Expert commentary on major open questions in photosynthesis research.

## Introduction

### (Written by Nancy A. Eckardt)

In the garden on a September morning, I was, once again, astonished to find that a tiny seed I planted a few months ago had produced large stems, leaves, and—seemingly daily—dozens of enormous fruit (also known as zucchini), literally out of thin air. This plant took carbon as CO_2_ directly out of the air and turned it into organic matter, and along the way, split water molecules to produce oxygen and stored energy, in one of the most remarkable and essential life processes: photosynthesis.

All oxygenic photosynthetic organisms have two photosystems (PS) known as PSI and PSII, which are embedded in thylakoid membranes and carry out light-driven electron transport. PSII absorbs light energy of wavelengths <680 nm to oxidize water into molecular oxygen (the “water-splitting” reaction), which contributes to the proton gradient needed to convert ADP to ATP, whereas PSI absorbs energy of longer wavelengths to generate high-energy electrons used to convert NADP^+^ to NADPH. All PSs have an array of light-harvesting antennae and mechanisms to dissipate excess energy under fluctuating light conditions. Many of the core reactions and features of the PSs are highly conserved among photosynthetic organisms. Research on the structure and function of the core components of the photosystems and thylakoid membranes often uses phototrophic microorganisms for the ease of maintaining cultures and isolating or purifying components. These include the green alga Chlamydomonas (*Chlamydomonas reinhardtii*), diatoms such as *Phaeodactylum* and *Thalassiosira*, and cyanobacteria such as *Synechocystis* and *Synechococcus*. With microorganisms, it is also possible to study in vivo and whole organism properties of photosynthesis without some of the many confounding processes present in land plants, such as stomatal conductance of CO_2_, water relations, and carbohydrate allocation (source–sink) dynamics.

A lot of photosynthesis research today is directed toward improving photosynthesis to create more climate-resilient crops and enhance crop yields in the face of global climate change (see the companion article in this issue by Croce et al. 2024). And yet, despite the long history of photosynthesis research (see e.g. Nelson and Junge 2015; Stirbet et al. 2020; Sharkey 2022) and significant advances we have seen in recent years (e.g. Croce and van Amerongen 2020; He et al. 2020; Perera-Castro and Flexas 2020; Bhowmick et al. 2023; Greife et al. 2023), we do not understand many aspects of photosynthetic energy conversion, such as details of its evolution, dynamics, and regulation.

In this commentary, a number of researchers working on fundamental aspects of photosynthesis pose and discuss what they view as the most compelling open questions in their areas of research. We begin at the reaction center and catalytic environment of the Mn cluster and move outwards to address questions related to light harvesting, photosystem evolution, thylakoid membrane dynamics, and alternative electron flow pathways. We have avoided questions related to Rubisco, as these have been covered in many recent reviews and perspectives (Carmo-Silva and Sharwood 2023, and references cited therein; Croce et al. 2024). Two final sections address the link between photorespiration and stomatal control and adaptive changes that make an enzyme suitable for its function in the C_4_ carbon-concentrating pathway present in important crop species.

## How does the catalytic environment of the Mn_4_CaO_5_ cluster accommodate and control the multielectron/multiproton reaction during water oxidation?

### (Written by Vittal K. Yachandra, Jan Kern, and Junko Yano)

**Figure 1.**
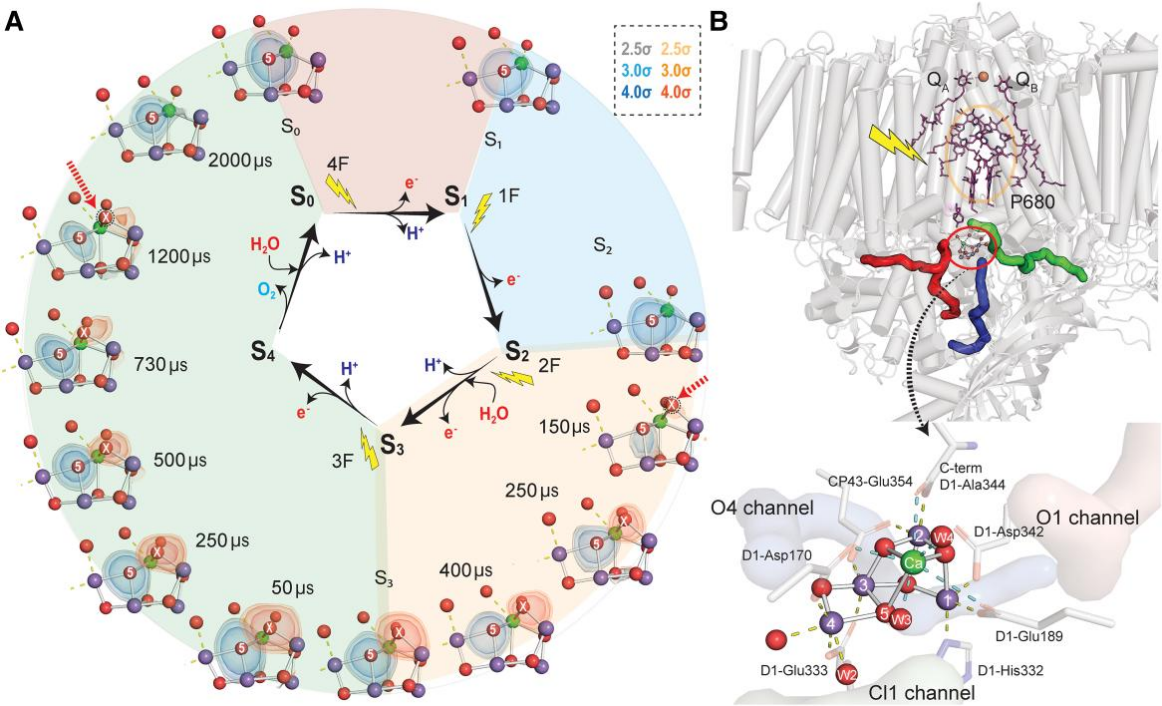
The Kok cycle and structure of PSII. **A)** The Kok cycle is shown in the middle, with the structures from XFEL crystallography of the S-states and at intermediate time-points between the S_2_ and S_3_, S_3_ and S_0_ states. O5 is shown in blue, and in red is the insertion of OX between Mn1 and Ca during S_3_ to S_0_ and its disappearance between the S_3_ and S_0_ transitions. The sigma value in crystallography is used for the scaling of the electron density; a higher value shows higher accuracy. **B)** On top is the structure of PSII, showing the electron transfer components, the OEC, and the proton and water channels (in red, blue, and green) in one monomer (left). The transmembrane helices and the helices in the extrinsic polypeptides are shown in color in the monomer on the right. Below this is the oxo-bridged Mn4Ca cluster in the S_1_ state in detail, with the water and other terminal ligands of Mn and Ca provided by the protein glutamate, aspartate, and histidine residues.

The model for photosynthetic water splitting proposed by Kok et al. (1970), based on the yield of dioxygen as a function of light flashes, has served as a blueprint for all subsequent work on the mechanism of the water-splitting reaction. This kinetics-based model proposed the advancement of the oxygen-evolving complex (OEC), the site where the oxidation of water occurs in photosystem II (PSII), through five intermediates known as the S-states (S_i_, i = 0 to 4). The absorption of four photons, one at a time, advances the OEC from S_0_ through S_4_, after which O_2_ is released. This model, called the Kok S-state cycle or clock (Fig. 1A), provided an elegant explanation for the coupling of the one-electron photochemistry occurring at the PSII reaction center to the four-electron redox chemistry occurring at the OEC. The working hypothesis was that upon the absorption of a photon, an oxidation equivalent is stored on the OEC, and after reaching the S_4_ state, the most oxidized intermediate, two water molecules are oxidized to form one molecule of O_2_. The S_0_, S_1_, S_2_, and S_3_ states are stable and can be trapped, but the S_4_ state is normally referred to as a short-lived or transient state that appears during the S_3_ to S_0_ transition prior to O–O bond formation and release of O_2_. The Kok cycle also entails the release of four protons and four electrons. The consensus is that the protons are released in a 1, 0, 1, 2 pattern for the S0 to S1, S1 to S2, S2 to S3, and S_3_ to [S_4_] to S_0_ transitions, and the electrons extracted from the donor OEC site by the PSII reaction center are transported via the electron transfer components to the quinones on the acceptor side.

Identifying the structure of PSII and the Mn-containing catalytic metal cluster of these intermediates, especially, during the S_3_-[S_4_]-S_0_ transition has been challenging and the subject of many studies (reviewed in (Wydrzynski and Satoh 2005; Nelson and Yocum 2006)) using diverse spectroscopic techniques, such as X-ray (Yachandra et al. 1996; Dau and Haumann 2008; Yano and Yachandra 2014), Fourier Transform Infrared Spectroscopy (FTIR; Debus 2015; Nagao et al. 2018), electron paramagnetic resonance (EPR; Lubitz et al. 2019), and X-ray crystallography (Umena et al. 2011). These studies revealed that the catalytic metal site consists of an oxo-bridged Mn_4_Ca hetero-nuclear cluster (Fig. 1B) that accumulates charge as it proceeds through the S-state cycle, reinforcing the hypothesis that the S_0_ through S_3_ transitions are primarily where the oxidizing equivalents are stored, with little or no specific chemistry related to the O–O formation from two water molecules. However, it was shown that during progression from S_0_ through the S_3_ state, the oxidation equivalents or the charge is not centered only on the Mn but more delocalized onto the ligands (Glatzel et al. 2004, 2013). There was no evidence for the presence of a peroxo or other intermediates, which are intermediate species one can expect for a water oxidation reaction. Intermediates such as metal-peroxo, -oxo, and -superoxo species have been detected in the study of inorganic water-oxidation catalytic systems. These data indicated that the entire chemistry involving water and the O–O bond formation occurs during the last step. This is the discharge/reduction step, where the most oxidized S_4_ state is reduced to S_0_ state (most reduced) with concomitant oxidation of water either in one fell swoop or in a stepwise manner, in which case it would proceed through the expected intermediates of water oxidation, with the most likely being two two-electron steps resulting in dioxygen via a transient peroxo intermediate.

The recent advent of X-ray free-electron laser (XFEL)-based room temperature crystallography and X-ray spectroscopy has had a profound influence on the structural studies and on the study of the mechanism of the water-splitting reaction (Kern et al. 2012, 2013, 2014; Suga et al. 2015). The structures of the S_0_, S_1_, S_2,_ and S_3_ states have been determined at ∼2 Å resolution (Fig. 1B). Furthermore, it is now possible to follow the structural changes between the S_2_ and S_3_states (Ibrahim et al. 2020) where the insertion of a new water occurs between Mn1 and Ca (likely as –OH after releasing one proton), and most importantly between the S_3_ and S_0_ states where the accumulated oxidizing equivalents are reduced and O_2_ is released (Fig. 1B) (Bhowmick et al. 2023).

Several events accompany the S_3_ to S_0_ transition that resets the Kok-clock to the S_0_ state, which includes the release of two protons, the release of dioxygen, and the recovery of one water at the OEC. The newly introduced ligand between Mn and Ca in the S_3_ state disappears during the S_3_ to S_0_ step, and this is related to the release of O_2_. The release of two protons signaled by changes in the gate region in the proton channel (Hussein et al. 2021) have been observed at two different time-points in the XFEL crystallography data (Bhowmick et al. 2023). Yet, it is not clear how exactly O–O formation and the recovery of the catalytic center takes place, due to the resolution of the current crystallography data that hinders accurate modeling of oxygen positions in the fraction of the changing population. The reduction process may create a longer lived intermediate, perhaps a peroxo species, which is two-electron reduced from the most oxidized S_4_ state. This is one step away from a further two-electron reduction of the OEC that results in the release of O_2_. We await structural studies at higher resolution.

The ability to visualize PSII structural changes through the catalytic process and understand the importance of the overall protein structure in the water-oxidation mechanism has also opened up new questions that are perhaps foundational to many multielectron catalytic reactions. How does the protein environment modulate the structure to neutralize the charge density changes on the OEC during catalysis? How does the water network, together with the amino-acid residues, facilitate proton transfer and substrate transport? How are the reaction kinetics affected by different environmental parameters such as pH and temperature? And how does the fundamental mechanism of the OEC remain the same through the diverse evolutionary tree (Hussein et al. 2023)? Recent advances in cryogenic electron microscopy in combination with site-specific mutations will likely contribute to answering some of these questions. While *Thermosynechococcus vestitus* (previously known as *T. elongatus*) has often been used for crystallography due to the availability of high-quality crystals, more species are being used for structural studies with cryogenic electron microscopy, and the structures from different organisms have been compared. Such comparisons can help to address questions about the fundamentally important design components for the water oxidation reaction in nature. In addition, the above-mentioned XFEL crystallography at room temperature, along with various spectroscopic methods, enables us to bridge structural information with spectroscopic data and provide the spatial and temporal sequence of events during the reaction. Thus, the study of the light-driven water oxidation reaction in PSII provides a unique opportunity to understand how the metal catalytic center and protein environment orchestrate and enable the multielectron/multiproton reaction, providing fundamental knowledge that goes beyond the water oxidation reaction.

## How do protein networks control light harvesting?

### (Written by Gabriela Schlau-Cohen and Dvir Harris)

**Figure 2.**
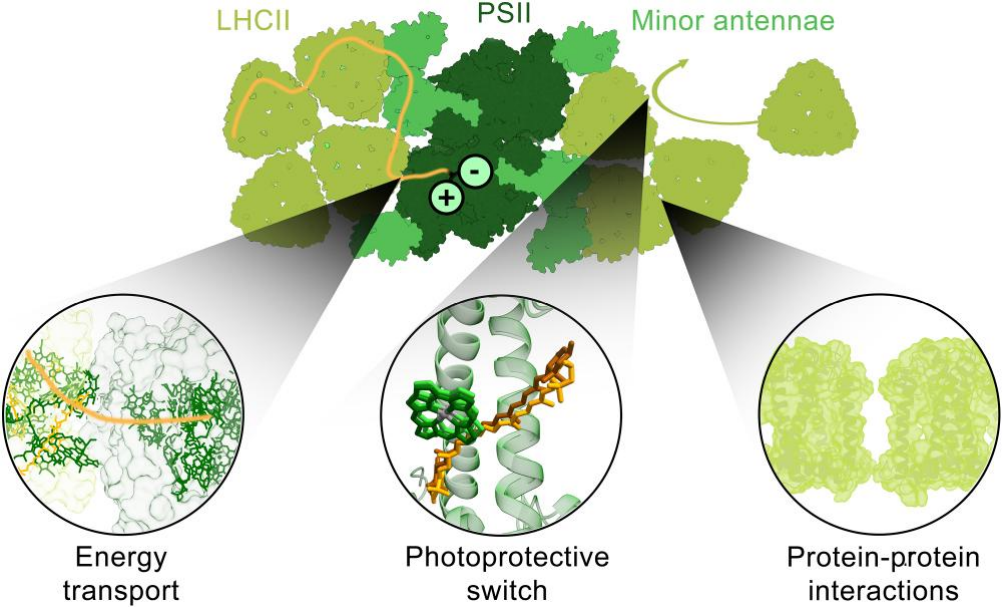
PSII from green plants. Chromophore-containing antennae capture and transport light energy (orange) to the PSII reaction center, where charge separation occurs (black). Under high light conditions, the local cellular environment and the organization of the protein network activate photoprotection within the antenna proteins, likely involving a switch of the photophysics of the embedded chlorophyll and carotenoids associated with different structural states (PDB: 5XNM). The protein network is formed through van der Waals interactions between proteins.

In photosynthetic light harvesting, a network of chromophore-containing antenna proteins absorbs light and transports photoenergy over distances of tens to hundreds of nanometers to reach reaction centers (Fig. 2). Despite the long distances, this transport can occur with near-unity quantum efficiency. At the same time, the efficiency is actively regulated by the network in a photoprotective response to light levels (Eberhard et al. 2008). The functionality of the network relies on the collective behavior of the constituent proteins. Yet studies have typically been limited to individual proteins, where relevant properties are often absent, or intact systems, where contributions from many proteins are combined to obscure behaviors of interest (van Grondelle and Novoderezhkin 2006). Resolution of the interactions and dynamics between proteins has been a long-standing challenge, leading to the major open question: *How do protein networks control light harvesting?* We discuss three facets of this open question.

### How is energy transferred between proteins?

The efficient transport of photoenergy through the antenna complexes to the reaction center is well established in photosynthetic organisms. Such long-distance transport emerges from a series of energy transfer steps within photosynthetic membranes. Cumulatively, these steps contribute to the hundreds of picoseconds timescale of solar energy conversion, whereas the relaxation time within individual proteins is approximately only one picosecond. The vast majority of previous experiments focused on individual proteins, even though transfer between proteins occupies most of the total time and is the key to long-distance energy transport (Fiebig et al. 2023). Despite its crucial importance, protein-to-protein energy transfer has been challenging to measure.

Several questions remain about how protein-to-protein energy transfer enables robust long-distance transport. In green plants, the energetic gradient of the protein network towards the reaction enter is nearly flat (Mirkovic et al. 2017). How a nearly flat gradient produces an effective directional energy flow remains unclear. Both the composition and organization of the protein network also change under light conditions. Energy transfer is highly sensitive to distance, and so even distance changes of a single nanometer can change the rate by an order of magnitude. Despite this sensitivity, the quantum efficiency of light harvesting changes minimally—less than 10%—with network organization (Fleming et al. 2012). Thus, how network reorganization impacts energy transport is not fully understood.

Despite the inherent challenges, several promising directions can now probe protein-to-protein energy transfer. Because of spectroscopic improvements, energy transfer rates have been extracted for spectrally shifted antenna proteins in membrane fragments or in vivo (Tiwari et al. 2018; Lüer et al. 2012). Alternatively, model membrane systems have been used to biochemically isolate antenna proteins for measurements of protein-to-protein energy transfer (Dai et al. 2018; Wang et al. 2023a, 2023b, 2023c). Finally, an integrative approach using single-molecule spectroscopy and cryogenic electron microscopy probed the heterogeneity associated with antenna to reaction center energy transfer (Harris et al. 2023). These recent technological advances enable investigation of the important and fundamental process of energy transfer between proteins.

### How does the network regulate photoprotection?

In oxygenic photosynthesis, excess energy can cause photooxidative damage to proteins. Under high light conditions, a drop in the lumenal pH activates a series of photoprotective processes, known as nonphotochemical quenching (NPQ). The fastest NPQ process is qE, the dissipation of excess energy as heat. The major antenna protein, light-harvesting complex II (LHCII), is a major site of dissipation for PSII (Schlau-Cohen et al. 2015; Nicol and Croce 2021), and its organization within the protein network changes, notably through the formation of clusters (Goral et al. 2012; Ruban and Johnson 2015). Recapitulating the physiological LHCII–LHCII interactions responsible for the functional switch has remained challenging, and so how these interactions regulate the dissipative pathways of photoprotection in vivo remains unclear.

Studies on aggregates and arrays of LHCII, which aim to emulate LHCII clustering, have shown enhanced dissipation, and a pH dependence reminiscent of the in vivo conditions (Barros and Kühlbrandt 2009; Petrou et al. 2014; Son et al. 2021). A dissipative photophysical pathway, chlorophyll-to-carotenoid energy transfer, was recently clearly resolved with the cluster and pH dependence expected for photoprotection. In recent in vivo measurements, the carotenoid photophysics correlated with NPQ (Park et al. 2019), suggesting that the photoprotective switch involves these important biomolecules. Such effects may be induced by LHCII clustering.

### What are the protein–protein interactions that govern network architecture?

In recent years, the organization of the protein networks has been revealed in exquisite detail (Wietrzynski et al. 2020; Opatíková et al. 2023; Feng et al. 2023). Advances in structural biology, including electron microscopy and tomography at cryogenic temperatures and atomic force microscopy, have provided snapshots that dramatically advanced our understanding. However, the interactions between the proteins that drive the formation of these networks and their reorganization in response to environmental stimuli remain unknown. While a small number of computational studies have investigated these interaction energies (Schneider and Geissler 2013; Mao et al. 2023), the well-established deviation between computational energies and experimental benchmarks (Keskin et al. 2016) means that new methods are needed to determine the thermodynamic driving forces behind protein networks. Recent experimental measurements of the interaction energies between LHCII revealed a net attraction of ∼−5 *k_B_T*, which represents an enthalpically dominated thermodynamic driving force behind LHCII clustering. Such studies capture the interactions behind the membrane organization of PSII from the perspective of equilibrium statistical thermodynamics, which has a long and rich tradition in biology (Manna et al. 2023).

In conclusion, structural and spectroscopic advances over the past decades built an understanding of the function and composition of the protein networks of photosynthetic light harvesting. In particular, the structure, organization, and dynamics of the constituent proteins have been revealed. The open questions sit at the interface between these proteins. Resolution of the interactions between proteins and how these interactions modulate function requires further improvements to our tools that characterize and manipulate these biosystems. Answering the central question—*How do protein networks control light harvesting?*—remains a grand challenge for the field.

## Why do diatoms have so many different light-harvesting proteins?

### (Written by Nicolas Herrmann and Claudia Büchel)

Vascular plants express an array of light-harvesting complexes (LHC) and the different functions of these LHCs as major or minor antennae for PSII, as PSI-specific antennae, or as mobile antennae in the context of state transitions are well described (Croce and van Amerongen 2020). Algae, by contrast, contain many more Lhc proteins (Lhc and LHC typically are used to denote the algal and vascular plant proteins, respectively), and one of the intriguing questions is the reason behind this diversity. Besides the well-studied green algae, there are many algal groups that use different pigments and even different proteins for light harvesting (Büchel 2015). Most prominent are the Stramenopiles, which are distantly related to green algae and vascular plants. These algae are derived from a so-called secondary endosymbiosis, where a eukaryotic host engulfed an organism related to red algae that was evolutionarily reduced to the chloroplast (Bhattacharya and Medlin 1995). The group of Stramenopiles with the best-studied light-harvesting systems are diatoms, unicellular organisms that are responsible for 20% of primary production worldwide (Field et al. 1998). Diatoms are found in many different habitats, ranging from biofilms to free-living marine or limnic planktonic forms. Marine diatoms are relatively easy to culture, and after whole-genome sequences as well as molecular tools for transformation became available, different species emerged as reference systems, i.e. the pennate diatom *Phaeodactylum tricornutum* and the centric diatoms *Thalassiosira pseudonana*, *Cyclotella meneghiniana*, and *Chaetoceros gracilis*.

**Figure 3.**
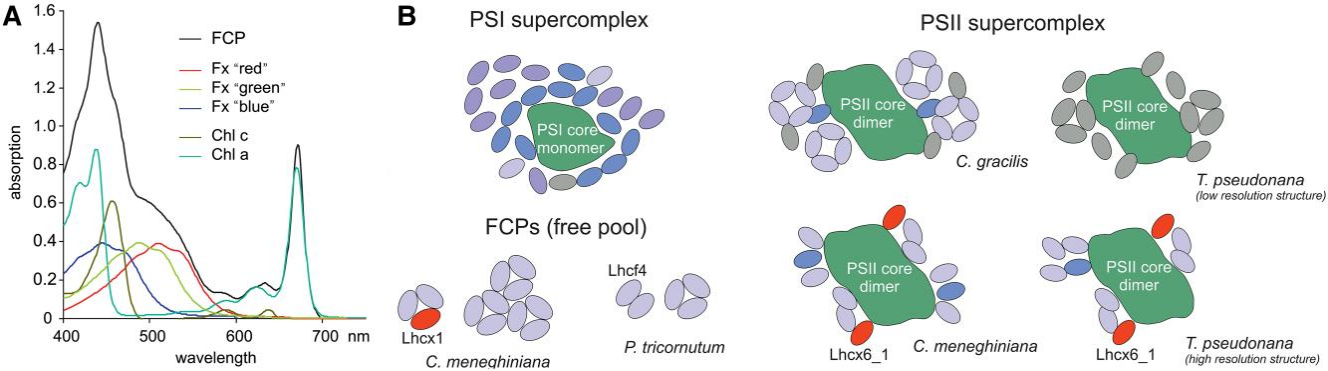
The FCP complex. **A)** Absorption spectrum of an FCP complex (black, FCPa from *C. meneghiniana*) and its pigments. Fx molecules are bound at different sites, absorbing more to the red wavelength range (Fx “red”), intermediate (Fx “green”), or to the blue (Fx “blue”). **B)** Scheme of FCP arrangement in PSI, PSII, and the free FCP pool of different species. Lhcx proteins are shown in red, other FCPs are colored according to their subfamily (light purple, Lhcf; blue, Lhcr; purple, lhcq; gray, unknown). For references, see text.

Diatoms possess a photosynthetic apparatus that is similar to that of vascular plants, including membrane-intrinsic antenna proteins of the Lhc family (Büchel 2015). However, their pigmentation is very different. Whereas chlorophyll (Chl) *a* is present in diatoms, Chl *b* is missing and only 2 to 3 molecules of Chl *c* are bound instead. The carotenoid fucoxanthin (Fx) is the main accessory pigment with up to seven molecules per protein of the eponymous Fucoxanthin-Chlorophyll Proteins (FCP). The Fx molecules are bound to various sites of FCP, inducing differences in absorption (Fig. 3A). Thus, FCP absorbs up to 585 nm, giving rise to the characteristic brown color of diatoms and optimizing photosynthesis in deeper water, where blue-green light dominates.

A wealth of spectroscopic data exists about the excitation energy transfer inside FCP complexes. Fx displays efficient excitation energy transfer to Chl *a* (Papagiannakis et al. 2005). The fastest transfer (∼30 to 60 fs) takes place between Chl *c* and Chl *a* (Butkus et al. 2015). Thus, excitation energy transfer between different Chl molecules is faster and FCP rely more on carotenoid absorption for light-harvesting than do Lhc of vascular plants. Nonetheless, these features are similar for all Lhc proteins from diatoms studied so far and do not shed light on a possible functional heterogeneity.

All diatom species contain a much higher number of expressed *Lhc* genes than found in vascular plants. *T. pseudonana* has more than 30 FCP proteins, *C. meneghiniana* has 23 FCPs, and *C. gracilis* and *P. tricornutum* have 22 and 32 FCPs, respectively (Kumazawa et al. 2022). PSI of diatoms is surrounded by monomeric FCPs, but the antenna is much larger than that of vascular plants, comprising ∼16 to 24 different FCPs in the case of *C. gracilis* (Nagao et al. 2020; Xu et al. 2020) (Fig. 3B). PSII structures are available from three centric species that differ tremendously in their FCP arrangement and composition, a diversity completely unknown in vascular plants. Whereas *C. gracilis* has three monomeric and two tetrameric FCP per core monomer (Pi et al. 2019), PSII of *C. meneghiniana* (Wang et al. 2023a, 2023b, 2023c) has only six FCP proteins. The high-resolution structure of *T. pseudonana* PSII shows one additional FCP (Feng et al. 2023) and is in contrast to the low-resolution structures, where three monomers and one trimer were modeled (Arshad et al. 2021). In addition, even the oligomeric state of FCP complexes found in the free pool of FCPs differs between species: for *P. tricornutum* Lhcf4 homodimers (Wang et al. 2019) and different trimers (consisting of the other FCP proteins including Lhcf4) (Gundermann et al. 2013) were reported, whereas analysis of *C. meneghiniana* revealed trimers and nonamers (Röding et al. 2018; Wang et al. 2023a, 2023b, 2023c). Oligomerization influences the pigment network and correspondingly, excitation energy transfer into the reaction centers. So far, no functional reason is known for this huge inter-species diversity.

To cope with rapid changes in light intensity while maintaining optimum photosynthetic energy flux, diatoms employ nonphotochemical quenching (NPQ), which is very fast and has a high capacity compared to NPQ of vascular plants (Ruban et al. 2004). NPQ is triggered by a high trans-thylakoid pH gradient under strong light and relies on a xanthophyll cycle that converts diadinoxanthin (Ddx) to diatoxanthin (Dtx) under excess light (Lavaud et al. 2002) and special FCP proteins called Lhcx. Diatoms contain many Lhcx proteins. Lhcx1 was demonstrated to be involved in NPQ in *P. tricornutum* (Bailleul et al. 2010) as well as in *C. meneghiniana* (Ghazaryan et al. 2016), but Lhcx1 cannot sense pH (Buck et al. 2021) and therefore the mechanism triggering NPQ remains enigmatic. Aggregation of FCPs containing Lhcx1 from the *C. meneghiniana* FCP pool reduced fluorescence in vitro and Dtx enhanced this fluorescence, which resembles NPQ in vivo (Gundermann and Büchel 2012). Domain swap experiments demonstrated the importance of a Trp residue of Lhcx1 that is close to the presumed Ddx binding site (Buck et al. 2021), hinting at a direct connection between Lhcx proteins and Ddx/Dtx. However, although the participation of Dtx in NPQ is well established, an in-depth spectroscopic analysis will be required to prove that Dtx that is bound to Lhcx1 participates in NPQ. The centric diatoms *T. pseudonana* and *C. meneghiniana* contain a special Lhcx protein, Lhcx6_1, which was found to be located in PSII complexes, harbors Dtx, and might be the second quenching site usually found in centric diatoms (Calvaruso et al. 2020, Grouneva et al. 2008). The sole presence of Dtx in an Lhcx structure does not prove its involvement in NPQ. Various abiotic stresses were shown to regulate the expression of the other three Lhcx proteins in *P. tricornutum* (Taddei et al. 2016), tuning NPQ. This functional diversification might help diatoms cope with variable environments.

Our understanding of the arrangement of FCPs around the photosystems, pigment organization, and excitation energy transfer has greatly improved in recent years. But why is FCP organization around PSII so diverse among the different species and what are the roles of the different FCP proteins? Involvement of FCPs in regulatory functions is the obvious idea to explain the huge diversity in and between species, but whether this explanation is true remains mostly enigmatic, as plasticity in the spatial arrangement of different FCPs remains unreported. And how precisely are the different Lhcx proteins involved in NPQ? Answering these questions will help us to understand the huge ecological and evolutionary success of diatoms.

## New perspectives on the evolution of the photosystems: Are the photosystems evolvable?

### (Written by Tom Dongmin Kim, Emma Chaloner, and Tanai Cardona)

How and when photosynthesis originated remain compelling questions in the Life and Earth Sciences. How photosystems, the multicofactor and multiprotein assemblies that power primary production, were put together from their constitutive parts and for what original function, are questions that still need answers. The photosystems are well over three billion years old and are potentially amongst the oldest enzymes (Oliver et al. 2023). On the one hand, that such endurance is possible suggests that photosystems have an in-built capacity for evolution and adaptability that enable photochemical processes to occur in as varied and dynamic conditions as can be found on Earth's photic zones, and on a planet that has gone through remarkable transformations over the eons. On the other hand, photosystems are highly conserved and slowly evolving enzymes, often perceived as somewhat immutable, even dubbed “frozen metabolic accidents” (Shi et al. 2005). Given that all photosystems share a common origin, evidence for the photosystems” evolvability is most conspicuously noted when comparing PSII and its unparalleled capacity to split water by generating some of the most oxidizing species in biology, with PSI, which has evolved to generate some of the most reducing ones.

These evolutionary considerations trigger a question that has received little explicit attention in the field of photosynthesis research: *are the photosystems evolvable?* Given that photosystems are biological systems, it is safe to assume that they are. However, the question raises a number of additional fundamental considerations about how photosystems have changed through time. For example, what are the molecular mechanisms that make the photosystems adaptable? How quickly can a photosystem gain a new function given adequate selective pressure? And, given selective pressures not found in nature, what new photochemical and catalytic properties could a photosystem evolve? We could go one step further: *Can photosystems be evolved in the lab?* We propose here that in an endeavor to answer these questions, we could gain new insights into why the photosystems evolved to be the way they are and open new pathways to greener chemical and biotechnological processes.

Photosystems are modular and this modularity is the basis for their capacity to remain adaptable. In the wild, this means that the photochemical or catalytic properties of a photosystem can be modified to respond to the environment by exchanging subunits, which work as replaceable modules. The better-known example of this is the fine-tuning of PSII energetics to perform optimally under different light intensities, and the most extreme example is the transformation of PSII from a water-oxidizing to a Chl *f*-producing enzyme (reviewed recently by Oliver et al. (2023)). In both cases, these functional alterations are achieved by replacing a standard D1 subunit for a variant form of D1. And in both cases, PSII evolved new photochemical or catalytic properties by acquiring modifications on a spare copy of a gene encoding the D1 subunit within the genome of cyanobacteria. Alterations of PSI by a similar mechanism are also known. For example, some cyanophages carry a set of PSI genes that are deployed upon infection of the cyanobacterium. In this adaptation, the standard PsaF and PsaJ subunits are replaced by a subunit encoded in the cyanophage's genome which is a fusion of the two (Sharon et al. 2009). This novel subunit has features that make the complex more promiscuous to protein electron carriers (Mazor et al. 2014). Another adaptation commonly observed in heterocystous cyanobacteria and their close relatives is the exchange of PsaB paralogs, which is likely to lead to a fine-tuning of the energetics of the complex in ways and conditions that are yet to be understood (Gisriel et al. 2023). Some strains encode up to four distinct PsaB versions. While the exchange of just a single subunit is sufficient to modify the photochemical and catalytic properties of the photosystems, there is no appreciable boundary as to the extent of change that can occur driven by sustained evolutionary pressures. A clear example of this is the far-red light photoacclimation response (Gan et al. 2014), which involves the swap of at least five subunits of PSII, and six of PSI, and the use of three distinct chlorophyll types to enable oxygenic photosynthesis under far-red light and in the absence of visible light.

The next step towards understanding the evolution of the photosystems is to evolve them in a laboratory setting and attempt to change their catalytic and photochemical properties purposefully. There is experimental evidence suggesting that the modularity of PSII can facilitate the application of directed evolution methods. For example, random mutagenesis of D1 was used to select for PSII complexes more tolerant to high light intensities (Narusaka et al. 1999), with tolerance to herbicides (Narusaka et al. 1998), and with tolerance to ionizing radiation (Rea et al. 2011). However, these studies stopped at a single round of mutagenesis and selection. In addition, directed evolution was used to successfully change the directionality of electron transfer of the purple bacteria reaction center (Faries et al. 2012).

**Figure 4.**
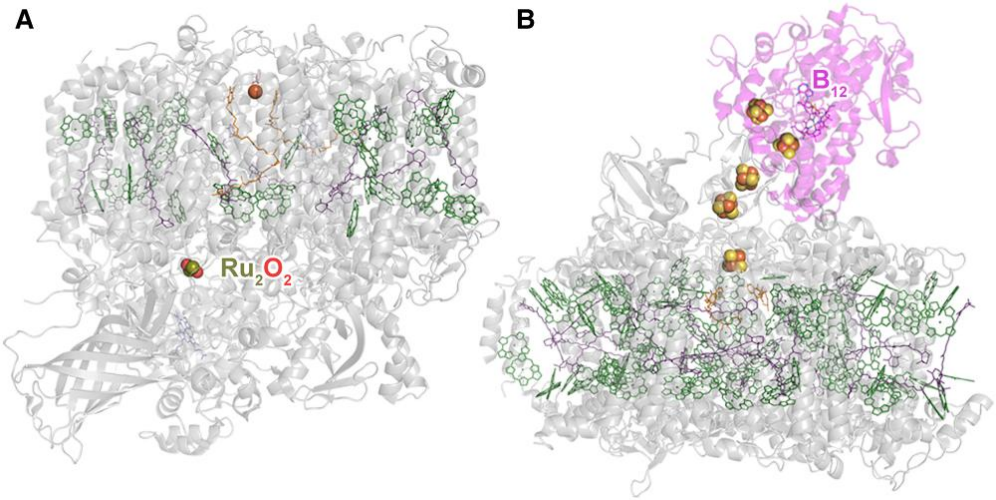
Conceptualization of evolved photosystems. **A)** A PSII evolved to bind a dinuclear Ru cluster (Ru_2_O_2_), which catalyzes a hypothetical specialized oxidative reaction. **B)** A hypothetical chimeric PSI fused with a reductive dehalogenase and evolved for optimal electron transfer to the catalytic site at a cobalamin cofactor (B_12_). Protein scaffolds of the photosystems are shown in transparent gray ribbons, and that of the reductive dehalogenase in magenta; chlorophylls are shown in green, carotenoids in purple, and iron–sulfur clusters are depicted with yellow and orange spheres. PSII structure was modified from PDB ID 3WU2, PSI from 1JB0, and reductive dehalogenase from 5M2G.

Evolving PSII could involve modifications of the electron donor side to change the nature of the water-oxidizing cluster and to enable access to other substrates (Fig. 4A). For example, to study one of the plausible transitional stages in the origin of PSII (Chernev et al. 2020), it may be possible to evolve a PSII that can efficiently oxidize Mn but cannot split water. Alternatively, a PSII could be evolved to decompose a complex organic pollutant in a sequential multielectron multiproton extraction process. The energetics of charge separation could be optimized for the specific catalytic demands imposed by the new substrates. There is evidence supporting the idea that P_D1_, one of the key redox chlorophylls of PSII that has a midpoint potential of about +1.2 V, is naturally tuned down by −0.14 V to compensate for electrostatic effects created by the Mn_4_CaO_5_ cluster (Ishikita et al. 2006), implying that the photochemical core could be pushed to generate even higher oxidizing potentials if desired. Evolving PSI could involve adding novel catalytic domains, which could be further optimized with directed evolution methods. In this sense, PSI could be engineered to transfer electrons from the terminal iron–sulfur clusters to the cofactors of the new domains, resulting in novel photobio catalysis (Fig. 4B). This concept has been demonstrated in the genetic fusion of the extrinsic subunits of PSI with hydrogenase enzymes (Appel et al. 2020; Kanygin et al. 2020; Wang et al. 2023a, 2023b, 2023c). The chimeras were all characterized as functional photosystems capable of light-driven H_2_ evolution. Such experiments, while opening new avenues for applications, would also test many existing rationales aimed at explaining photosystem structure and function. For example, what are the exact energetic tradeoffs between photochemical efficiency and photoprotection? Thus, if oxygen were not to be a reaction by-product of a hypothetical evolved PSII oxidizing a new substrate, could energy that is sacrificed by the reaction center to prevent back-reactions be redirected towards improving turnover efficiency? If that were the case, then, what would be the maximum rate of water oxidation of PSII, i.e. if the system had not needed to evolve into its bioenergetics protective mechanisms against the production of reactive oxygen species? By exploring these questions, a positive feedback loop can be created between research aimed at understanding photosystem evolution and developing new photosystem-based technologies for better biocatalysis.

To conclude, two specific questions can be asked to guide new research. First, what are the limits of photosystem evolvability? The answer to this question will help us understand the limits of light-dependent life on Earth and extrasolar planets in habitable zones. It can also help us understand why life came to be the way it is. Second, how can novel light-driven enzymes, featuring customized photochemical and catalytic properties, contribute to making the chemical and biotechnological industries more sustainable? This question is an invitation to a new generation of photosynthesis researchers to use their creativity in exploring how new photosystems could be harnessed as solutions to some of the global challenges we face.

## Thylakoid membrane dynamics: How are protein biogenesis and mobility orchestrated in this densely packed system?

### (Written by Conrad Mullineaux)

Thylakoid membranes in chloroplasts and cyanobacteria are densely packed with protein complexes. They present a crowded environment, not only in the plane of the membrane but also in the third dimension, where parallel membrane surfaces may be tightly appressed, or in close proximity and sandwiching a dense mass of soluble protein. Dense packing of the system allows a high concentration of pigment-protein complexes, which is necessary for the efficient absorption of sunlight. However, it creates challenges for other aspects of membrane function, including regulatory re-organization, diffusion of mobile electron carriers, and the biogenesis of protein complexes, which could all be impeded by the crowded environment in and around the thylakoids (Kirchhoff 2014a).

**Figure 5.**
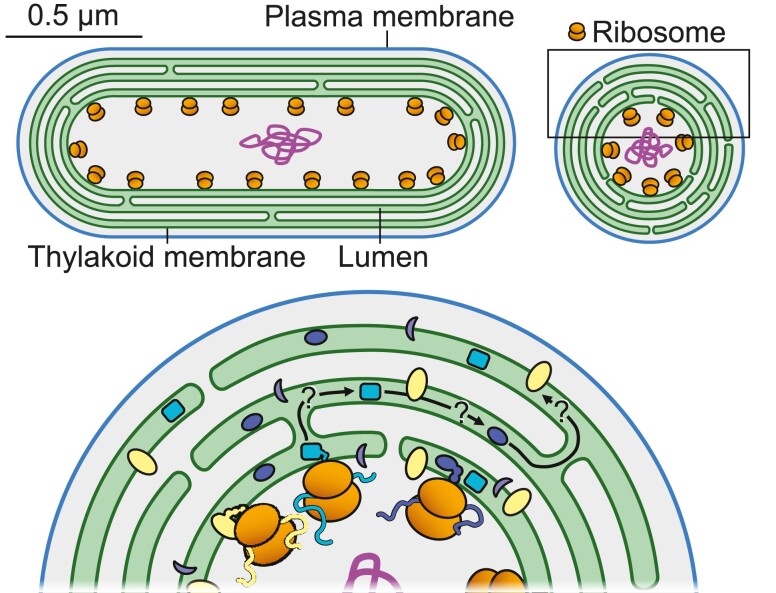
The photosynthetic protein biogenesis problem, illustrated by a schematic cross-section of a cell of the cyanobacterium *Synechococcus elongatus*. Translation of membrane-integral photosynthetic proteins occurs only at the proximal thylakoid surface facing the central cytoplasm and the nucleoid. How do new complexes get to the more distant parts of the thylakoid system?

The translation and membrane insertion of membrane-integral protein complexes invariably seem to occur in specialized regions of the membrane that may be quite remote from much of the mature functioning thylakoid surface. This likely arises because the sheer bulk of the ribosomes excludes them from most of the crowded thylakoid system. In cyanobacteria, ribosomes are found only at the proximal thylakoid surface adjacent to the central cytoplasm and are therefore excluded from about 90% of the thylakoid system (Fig. 5; Rast et al. 2019; Mahbub and Mullineaux 2023). mRNAs encoding membrane-integral thylakoid proteins are found at the same membrane surface (Mahbub et al. 2020; Mahbub and Mullineaux 2023). In chloroplasts of the green alga Chlamydomonas, translation is heavily focused on a specialized “T-zone” surrounding the pyrenoid (Schottkowski et al. 2012). In vascular plant chloroplasts, ribosomes are excluded from the tightly appressed grana membrane surfaces, but ribosomes also appear very scarce across large areas of the stromal lamellae, being concentrated at the most peripheral regions of the thylakoid that have unimpeded access to the chloroplast stroma (Shimoni et al. 2005).

If translation at the thylakoid is highly localized, it raises the question of how newly synthesized protein complexes can find their place in the mature thylakoid membrane. Thylakoid membranes seem to be more interconnected than is often apparent at first sight. For example, the thylakoids of the cyanobacterium *Synechococcus elongatus* approximate a set of nested cylinders (Fig. 5), but electron tomography revealed membrane bridges that interconnect the whole system (Nevo et al. 2007). So, it is likely that there is a continuous membrane surface connecting every region of a thylakoid to a translation zone, but the problem of restricted protein diffusion in the crowded thylakoid membrane environment remains—is it feasible for a newly synthesized complex to diffuse from the site of its translation to the furthest parts of the thylakoid system? Taking *S. elongatus* as an example, Fluorescence Recovery after Photobleaching (FRAP) measurements on GFP-tagged photosynthetic complexes suggest long-range lateral diffusion coefficients of the order of 10^−10^ cm^2^s^−1^ (Casella et al. 2017). Such a diffusion coefficient would permit a complex to diffuse on average 1 *µ*m from its starting point in 25 s, however for radial diffusion from the proximal to the distal layers of the thylakoid system the problem is hugely compounded by the need for the complex to encounter one of the very occasional bridges that connect the concentric thylakoid membrane layers (Nevo et al. 2007). There is an analogous problem in chloroplast appressed grana membranes, where FRAP measurements suggest a limited mobile pool of chlorophyll–protein complexes with a diffusion coefficient of about 5 × 10^−11^ cm^2^s^−1^ (Kirchhoff et al. 2008), but there are restricted connections between the appressed membranes and the stromal lamellae (Mustárdy et al. 2008). Computational modeling combined with high-resolution data on 3D thylakoid ultrastructure will be needed to define the extent of the problem.

If, as seems likely, movement of newly synthesized complexes to the furthest parts of the thylakoid system is slow, then what are the solutions and what are the functional implications? Taking *S. elongatus* (Fig. 5) as an example, two extreme scenarios could be envisaged for the incorporation of new complexes into the expanding thylakoid membrane as the cell grows. In the first scenario, new complexes stay close to their site of translation. As the cell grows and more complexes are synthesized, the parts of the proximal membrane surface mature into fully functional thylakoid. New areas of biogenic membrane surface are generated to replace them, so that new thylakoid is created at the proximal side of the thylakoid system and older areas of thylakoid are gradually pushed towards the cell periphery as the cell grows. One piece of evidence against this idea is that in *S. elongatus* cells that are regenerating their thylakoid system after growth in high light, new thylakoid membrane sacs seem to appear at the distal edge of the system between the existing thylakoids and the plasma membrane (Huokko et al. 2021). These new membranes must presumably be populated by protein complexes migrating from the proximal side of the system. The second scenario assumes that the proximal thylakoid membrane surface remains as a biogenic surface throughout, and complexes migrate out of it as they are assembled. It seems that this idea must apply at least to the replacement of the D1 subunit of PSII, which is rapidly turned over in high light (Komenda et al. 2012). Newly translated D1 proteins must surely be needed for repair of damaged PSII complexes throughout the thylakoid system. Interestingly, there are indications that the mobility of chlorophyll–protein complexes increases following high light exposure, in both chloroplasts (Goral et al. 2010) and cyanobacteria (Sarcina et al. 2006).

Both scenarios outlined above would predict gradients in thylakoid membrane protein composition depending on their distance from the nearest translation site. Repair and regulatory adjustments to the population of photosystems (e.g. (Dietzel et al. 2008; MacGregor-Chatwin et al. 2022) might take effect at different speeds in different regions of the membrane, with strong functional consequences. Understanding the consequences will require progress in cell biological approaches to thylakoid biogenesis, to complement the remarkable advances from biochemical and structural approaches (e.g. (Zabret et al. 2021)). Electron tomography has provided new insight into the 3D architecture of thylakoids, as in (Shimoni et al. 2005; Nevo et al. 2007; Engel et al. 2015; Rast et al. 2019), and this should be coupled with fluorescence microscopy to probe membrane dynamics. Techniques such as structured illumination microscopy, as in (Wood et al. 2018), will allow higher resolution than was previously achievable. It would be exciting to develop fluorescent labeling methods that distinguish between newer and older photosystems. This could reveal the flux of the membrane system over time, as the cell or organelle expands and divides. How do the dynamics of the membrane and the locations of membrane protein production constrain photosynthetic function, and how much do they restrict acclimation to new conditions? These will be overarching research questions for the next years.

## How fluid are thylakoid membranes?

### (Written by Wojciech Wietrzynski and Benjamin D. Engel)

One of the most intriguing characteristics of thylakoid membranes is their diverse molecular architecture (Perez-Boerema et al. 2024). In cyanobacteria, as well as the chloroplasts of red algae and glaucophytes, thylakoids are decorated with bulky phycobilisome light-harvesting antennae, which space the membranes apart and prevent stacking interactions. Here, PSII has been observed to form linear arrays, bound underneath rows of phycobilisomes (Rast et al. 2019; Li et al. 2021a, 2021b). PSI has been observed to bind alongside these PSII arrays in red algae (You et al. 2023), or in cyanobacteria to form trimers or tetramers occupying separate domains within the nonstacked thylakoids (MacGregor-Chatwin et al. 2017).

In contrast, thylakoid architecture in the chloroplasts of green algae and plants can generally be subdivided into appressed and nonappressed (stacked and unstacked) domains (Mustárdy et al. 2008). These chloroplasts lack phycobilisomes and instead rely on light-harvesting complexes (LHCs) and related membrane-integral antenna proteins, which do not space the thylakoid membranes apart. In fact, there is evidence that interactions between LHCII proteins contribute to membrane stacking in plants (Standfuss et al. 2005; Daum et al. 2010; Zeno et al. 2022). Stacking enforces lateral heterogeneity in thylakoid membranes: PSI and ATP synthase populate the nonappressed domains, whereas PSII is restricted to the appressed domains, and only cytochrome *b*_6_*f* is abundant in both domains (Andersson and Anderson 1980; Wietrzynski et al. 2020). This segregation of components prevents energy spillover from PSII to PSI, and has deep implications for the regulation of photosynthesis. Balancing of PSI and PSII excitation via migration of LHCII between domains (state transitions; Allen and Forsberg 2001), repair of PSII complexes damaged by photoinhibition (Barber and Andersson 1992), and integration of newly synthesized proteins into the appressed membranes would all require the exchange of protein complexes between the stacked and unstacked thylakoid regions. Therefore, to respond to varying environmental conditions that chloroplasts experience throughout a typical day, thylakoids should undergo some structural changes, at least at the scale of single protein complexes moving between membrane domains.

But how fluid are these thylakoid membrane domains? In other words, how mobile are the protein complexes and lipids within the plane of the membrane under short timescales? Do the components freely mix like a liquid, or are they more static like a gel? While plastoquinone must diffuse between PSII and cytochrome *b*_6_*f*, it is unknown whether the larger photosynthetic complexes freely move around within their resident membrane domain or are fixed in space and experience only small oscillations (Kirchhoff 2014a). On the one hand, the low saturation of thylakoid lipids should facilitate the diffusion of protein complexes in the membrane; on the other, mobility might be restricted by molecular crowding from the high protein concentration (thylakoids are ∼70% protein [Kirchhoff et al. 2002]) as well as electrostatic interactions between stacked thylakoids (Standfuss et al. 2005). Can current technology measure the degree of mobility for thylakoid protein complexes in vivo? And would changes in this mobility impact cellular physiology and photosynthesis? Does it matter whether thylakoid membranes are more fluid or more static?

Two electron microscopy (EM) techniques can directly visualize individual protein complexes within thylakoid membranes in vivo. Freeze-fracture EM has been used for decades to quantify the organization of photosynthetic complexes within fractured membrane planes (Ojakian and Satir 1974; Charuvi et al. 2016). More recently, cryo-electron tomography has extended this visualization to both sides of thylakoid membranes within the cell, providing a clear view of the lateral heterogeneity between membrane domains (Wietrzynski et al. 2020). However, these EM approaches can only provide frozen snapshots of protein complex organization, begging the question of how mobile these complexes are. Fluorescence recovery after photobleaching (FRAP) can provide information on protein mobility, and it has been used to determine that PSII complexes do not rapidly diffuse between separate grana stacks (Kirchhoff et al. 2008), which is consistent with lateral heterogeneity. However, the resolution of this technique is far too limited to follow the mobility an individual PSII complexes within an appressed grana membrane. Spectroscopy can detect in vivo changes in the general ordering of thylakoid proteins, but it provides no spatial information on the movement of complexes. Only high-speed atomic force microscopy (AFM) combines the spatial resolution to map single complexes in a membrane with the temporal resolution to track their mobility (Clausen et al. 2014; Onoa et al. 2020). However, AFM is limited to measuring the topology of isolated membranes, which may not reproduce the thylakoid organization and fluidity found inside a living cell. Unfortunately, no single technique in use today can track the short-timescale mobility of individual protein complexes within native thylakoids.

**Figure 6.**
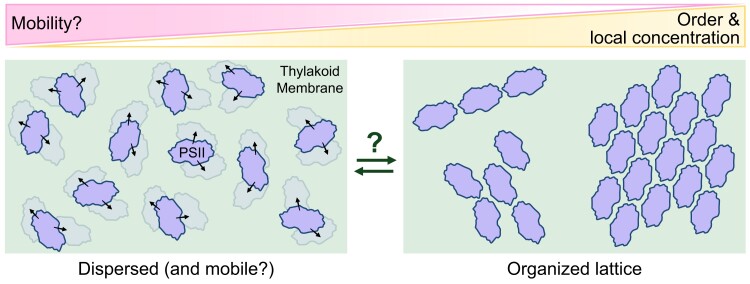
Does PSII organization and mobility change in vivo? Schematic illustrating the dispersed and pseudocrystalline lattice arrangements of PSII (blue), which could potentially modulate an inverse relationship between local PSII concentration and mobility. Whether such a transition in PSII organization is physiologically relevant in vivo remains to be explored.

Observations of PSII organization provide hints that this photosynthetic complex may experience changes in its mobility, and also underline the potential differences between isolated and native membranes. In cyanobacteria and red algae, PSII complexes bound along linear arrays of phycobilisomes must experience some restriction in their degrees of freedom. In isolated thylakoids of plants and diatoms, PSII has been observed in both a dispersed organization and arranged in two-dimensional pseudocrystalline lattices (Fig. 6) (Staehelin 1976; Dekker and Boekema 2005; Kirchhoff et al. 2007; Daum et al. 2010; Sznee et al. 2011; Levitan et al. 2019). These PSII lattices would further restrict the degrees of freedom, and they also interact with lattices in neighboring membranes to induce stacking of isolated thylakoids (Daum et al. 2010), limiting the mobility of PSII in two appressed membranes. In vivo, only the dispersed organization of PSII has been observed to date, with the exception of PSII lattices within dehydrated resurrection plants (*Craterostigma pumilum*) (Charuvi et al. 2015).

What would happen to PSII-related processes when the complexes are organized in a lattice within appressed thylakoids? How would the lattice affect plastoquinone diffusion to and from PSII? Are PSII able to leave the lattice when damaged? Are initiators (e.g. kinases; Depège et al. 2003) and effectors (e.g. proteases; Lindahl et al. 2000) located in the stroma able to reach them? Do PSII lattices receive less excitation from LHCII? If PSII lattices are physiologically relevant, perhaps a transition between mobile dispersed PSII and the static lattice configuration can help chloroplasts resist particularly harsh environmental conditions, such as dehydration of resurrection plants. The thylakoids may be locked in a nonphotosynthetic state that can activate again upon rehydration. Could such a change in thylakoid fluidity occur locally as a photoprotective mechanism to regulate excess excitation or to sequester subpopulations of damaged PSII? Due to the technical challenge of observing PSII dynamics in vivo, exploring these questions will require approaches that integrate structural observations, biophysical measurements, physiology, and molecular simulations.

## How does alternative electron flow contribute to the maintenance of robust photosynthesis?

### (Written by Arthur R. Grossman)

Light absorption drives photosynthetic electron transport and the synthesis of reductant and ATP to energize CO_2_ fixation, growth, and other anabolic processes. However, photosynthetic antennae often absorb excess excitation energy, especially upon exposure of plants to high/fluctuating light intensities or stress (Saroussi et al. 2017). To cope with excess absorbed light energy, photosynthetic organisms have evolved mechanisms to dissipate this energy as heat through nonphotochemical quenching (NPQ), including energy-dependent quenching (qE), state transitions (qT), photoinhibition (qI), and zeaxanthin-dependent quenching that does not require a high ΔpH across the thylakoid membranes (qZ) (Niyogi and Truong 2013).

**Figure 7.**
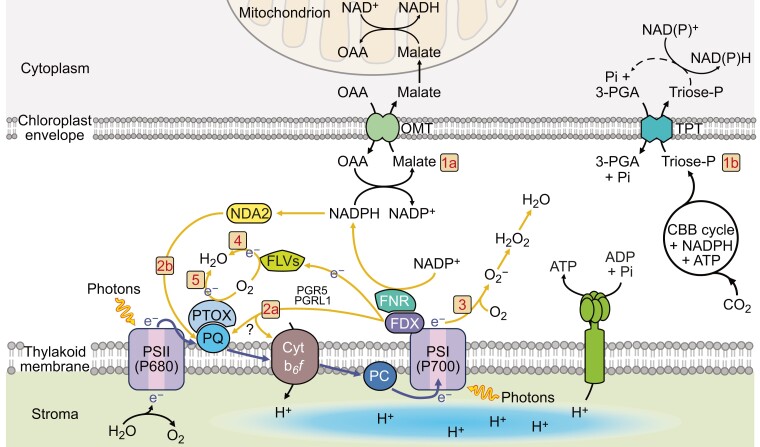
Diagram of photosynthetic electron transport in Chlamydomonas, two major pathways for exporting fixed carbon and/or reductant from chloroplasts, and alternative pathways for electron flows (AEF). Linear photosynthetic electron transport involves excitation of reaction centers (PSI, PSII) and extraction of electrons from H_2_O by the PSII O_2_ evolving complex. Extracted electrons pass through PSII reaction centers, the plastoquinone (PQ) pool, Cytochrome *b*_6_*f* (Cyt), plastocyanin (PC) and to PSI where they are used to generate reduced ferredoxin (FDX) and NADPH; the ATP synthesized by the ATP synthase (fueled by proton gradient across thylakoid membranes) and the NADPH are used to drive CO_2_ fixation by the Calvin-Benson cycle. Major steps in these pathways are numbered in orange boxes. Fixed carbon and reductant are exported from chloroplasts by various shuttles; two major shuttles involve oxaloacetate/malate and triose-P/Pi exchange (OMT and TPT, respectively, 1a and 1b). Additionally, reducing electrons generated on the acceptor side of PSI or PSII can be routed through other AEF pathways (corresponding to 2 to 5) which include cyclic electron flow through both PGR5/PGRL1 (2a) and NDA2 (2b) pathways, the Mehler reaction (3) in which PSI-derived electrons are used to reduce O_2_ and the ROS generated can be converted to H_2_O through superoxide dismutase and catalase/ascorbate peroxidase, pseudocyclic electron flow (4) in which electrons can be used to reduce O_2_ to H_2_O through FLV flavodiiron proteins, and plastoquinol terminal oxidase (PTOX) catalyzed reduction of O_2_ (5) associated with the acceptor side of PSII. Major pathways associated with AEF are depicted with orange arrows while black arrows indicate linear electron flow.

Photosynthetic electron transport involves linear electron flow, which generates ATP and reducing power, and alternative electron flows (AEF), including cyclic electron flow and H_2_O-to-H_2_O cycles in which O_2_ is reduced to H_2_O (Fig. 7). Linear electron flow does not generate enough ATP to sustain photosynthesis, a deficit made up by the various AEF pathways. Additionally, AEF deposits protons in the thylakoid lumen that can help establish NPQ, impact the rate of linear electron flow, protect PSI from damage under high/fluctuating light (see the section below by Burlacot), and help balance the ATP:NADPH ratio, which can impact PSI donor and acceptor side regulation (Munekage et al. 2004; Allahverdiyeva et al. 2015a, 2015b). AEF reactions include (i) Mehler-type, (ii) NADPH:flavin oxidoreductase (FLV) catalyzed O_2_ reduction, designated pseudocyclic electron flow, (iii) plastoquinol terminal oxidoreductase (PTOX) activity, and (iv) chloroplast-to-mitochondria electron flow, which shuttles electrons between the organelles. The biochemical shuttles important for the flow of reductant between the chloroplast and mitochondria translocate oxaloacetate/malate (OMT; Fig. 7, #1a), malate/aspartate (Dao et al. 2022), and triose-P/Pi (TPT) (Fig. 7, #1b) (Huang et al. 2023), coupled with redox exchange reactions catalyzed by malate dehydrogenase (MDH) and glyceraldehyde 3-P dehydrogenase (GAPDH). We describe basic features of these pathways, with a focus on Chlamydomonas, and highlight some major unanswered questions about their role(s) in maintaining robust photosynthesis.

### Cyclic electron flow

Cyclic electron flow (Fig. 7, #2a, 2b) directs reducing equivalents from the PSI acceptor to the donor side, enabling proton deposition in the thylakoid lumen and the use of the proton gradient for ATP synthesis without generating reducing equivalents. Most photosynthetic organisms use two cyclic electron flow pathways, one involving the proton gradient generation 5 (PGR5) and the PGR-Like 1 (PGRL1) proteins (DalCorso et al. 2008), and the other the plastid Type I NDH (NADPH:flavin oxidoreductase) complex in vascular plants (Joet et al. 2001) or Type II NDH, designated NDA2, in green algae (Desplats et al. 2009). The Type I NDH has 11 chloroplast-encoded and >19 nucleus-encoded subunits and is similar to the bacterial/mitochondrial NADH:UQ respiratory complex (complex 1) (Peltier et al. 2016); it likely uses reduced ferredoxin (FDX) as the electron donor (Yamamoto et al. 2011). The PGR5/PGRL1 pathway also appears to involve PetO, FNR, and ANR2 (Buchert et al. 2018) that may function in a cyclic electron flow supercomplex (Joliot et al. 2022). Tobacco (*Nicotiana tabacum*) and Arabidopsis (*Arabidopsis thaliana*) with lesions in genes encoding chloroplast NDH subunits do not exhibit strong phenotypes under mild conditions (Shikanai et al. 1998). The Chlamydomonas Type II NDA2 transfers electrons from NAD(P)H to the PQ pool and becomes highly active during N deprivation (Saroussi et al. 2016).

### Mehler reaction

The Mehler reaction (Fig. 7, #3) involves noncatalyzed O_2_ reduction using PSI-derived electrons (Asada 1999). It can generate reactive oxygen species (ROS), including superoxides (O_2_^−^) and hydrogen peroxide (H_2_O_2_) that can cause damage to various molecules of the cell, or the O_2_^−^ can be converted to H_2_O by the sequential activities of superoxide dismutase and ascorbate peroxidase/catalase. The Mehler reaction can also generate a ΔpH across thylakoid membranes (Makino et al. 2002), although it is unclear how prevalent this reaction is in Chlamydomonas.

### Pseudocyclic electron flow

Pseudocyclic electron flow (Fig. 7, #4) involves electron transfer to PSI-associated flavodiiron proteins (FLVs), likely through FDX (Sétif et al. 2020), which are thought to directly reduce O_2_ to H_2_O (Chaux et al. 2017). This reaction occurs in green algae, cyanobacteria, mosses, liverworts, and gymnosperms, but not in angiosperms, and is especially important immediately after dark acclimation when the Calvin-Benson cycle is not activated, and under fluctuating light conditions (Allahverdiyeva et al. 2013). A more detailed discussion of the flavodiiron proteins is given in the section below by Allahverdiyeva et al.

### Plastoquinol terminal oxidoreductase

PTOX, associated with chlororespiration (Fig. 7, #5), uses electrons from the PQ pool to reduce O_2_ to H_2_O (Peltier et al. 2010). These electrons can come directly from PSII or from NADPH to the PQ pool via the NAD(P)H-PQ reductase; Type I NDH in plants and NDA2 in green algae (e.g. Chlamydomonas). Potential roles of PTOX include protection of cells during fluctuating light (Nawrocki et al. 2019) and PQ pool over-reduction, which can elicit PSII charge recombination and the generation of singlet O_2_. Chlamydomonas has two PTOX isoforms (PTOX1, PTOX2) (Houille-Vernes et al. 2011), with PTOX1 likely involved in regenerating oxidized PQ for phytoene desaturation (Foudree et al. 2012).

### Chloroplast-to-mitochondria electron flow

The transfer of reducing equivalents between chloroplasts and mitochondria, here referred to as chloroplast-to-mitochondria electron flow (Fig. 7, #1a,1b), is also integral for controlling cellular energetics and redox conditions in the light, and for sustaining dark anabolic processes (Dang et al. 2014). Chloroplast–mitochondria interactions were suggested in studies using inhibitors of mitochondrial electron flow and mutants in chloroplast ATP synthase (Lemaire et al. 1988) and respiration (Cardol et al. 2009). The integration between chloroplast and mitochondrial electron flow (He et al. 2023) helps coordinate the two pathways, balancing the production of reductant and ATP, regulating the metabolism of phosphate, nitrogen, and carbon compounds, and potentially managing accumulation of ROS to help sustain chloroplast electron transport and CO_2_ fixation. Major translocators for chloroplast-to-mitochondria electron flow have been proposed to involve OMTs (malate/oxaloacetate, malate/aspartate) and TPTs. Plants and algae have multiple OMTs and TPTs, which are bidirectional transporters that function based on mass action and can move carbon and reductant out of chloroplasts (as these metabolites accumulate in the stroma), delivering them to other cellular compartments, including mitochondria. Reductant delivered by these transporters to mitochondria can serve as substrate for respiratory electron transport and reduction of O_2_ through cytochrome oxidase or alternative oxidases (AOX), which are present in both plant and algal mitochondria. Inhibitor studies (myxothiazol, an inhibitor of the mitochondrial cytochrome *bc*_1_ complex, and salicylhydroxamic acid, an inhibitor of the mitochondrial alternative oxidase) also suggest that the AOX pathway may generate some chemical bond energy/ATP that can support photosynthetic CO_2_ fixation through the formation of electrochemical potential (although AOX does not pump protons), but not as much as the cytochrome oxidase pathway (Peltier et al. 2023). The AOX pathway is cyanide insensitive, enables continued operation of glycolysis and the TCA cycle under stress conditions when there is restricted electron flow through cytochrome oxidase, balances C:N, ATP:ADP, NAD(P)H:ATP ratios, and limits hyper-reduction of the respiratory chain and accumulation of ROS (Rogov et al. 2014; Kaye et al. 2019).

While OMTs are thought to mainly shuttle reducing equivalents between chloroplasts and other cellular compartments, TPTs may mainly export fixed carbon synthesized in chloroplasts (Huang et al. 2023). Chloroplast TPTs can transport triose-P [glyceraldehyde 3-P (GAP), dihydroxyacetone-P (DHAP), and 3-phosphoglycerate (3-PGA)] in a counter exchange for cytosolic inorganic phosphate (Pi), which resupplies the chloroplast with Pi, allowing for continued photosynthetic electron flow and CO_2_ fixation. TPTs are part of the pPT plastid translocator family that can serve as antiporters of phosphorylated C_3_, C_5_, or C_6_ compounds with Pi (Flügge et al. 2003; Bockwoldt et al. 2019). In plants, trioses exported from chloroplasts are used to synthesize sucrose and other metabolites and fuel respiratory activity. Plants also harbor other pPT subfamilies, including those for glucose 6-P (GPTs) (Kammerer et al. 1998), xylulose-P (pentose-P) (Eicks et al. 2002), and phosphoenolpyruvate (PEP) (PPTs) (Fischer et al. 1997); PEP can be imported into C_3_ plant plastids and used as substrate to fuel fatty acid biosynthesis or the shikimate pathway, and exported from C_4_ plant plastids (Streatfield et al. 1999; Prabhakar et al. 2010). In some plants, diminished TPT levels do not yield a strong phenotype because the loss of plastid TPT activity can be compensated for by accumulation of a transitory starch pool that can be rapidly degraded (Hausler et al. 1998, Walters et al. 2004) to products (e.g. hexose-P) exported from chloroplasts and used in other cellular compartments. Chlamydomonas TPT3 was shown to likely be most active in routing triose-P out of chloroplasts; *tpt3* null mutants exhibited aberrant photosynthesis and highly reducing conditions in chloroplasts (Huang et al. 2023).

### Contributions of AEF pathways

The relative importance of the different AEF pathways in generating the ATP needed to sustain CO_2_ fixation has been evaluated using electron transport inhibitors and mutants. Cyclic electron flow, pseudocyclic electron flow, and chloroplast-to-mitochondria electron flow activities were quantified in Chlamydomonas and shown to have compensatory activities, with each able to provide a large fraction of the energy needed to sustain photosynthesis (Peltier et al. 2023); the most energetically efficient pathway was chloroplast-to-mitochondria electron flow. However, using mutants and pathway inhibitors to quantify individual pathway activities may not accurately reflect in vivo wild-type activities since a loss of any of these pathways in vivo may alter the contributions of the remaining pathways, and in some cases the inhibitors may not be completely penetrant.

### Questions concerning integration of AEF activities

Photosynthetic electron flow and energy transfer are highly complex processes (Fig. 7) and there is ongoing debate about the importance of the different AEF pathways. Electron flow and energy transfer through these pathways largely occur through metabolic flux driven by mass action. This means that blocking one “road” can increase flux through another. Organisms can make adjustments by modifying gene expression or other regulatory phenomena. Some of the unknown questions include the following.

1. Can we measure the precise contributions of each AEF pathway in vivo without disrupting (mutants, inhibitors) the system?
2. What conditions govern the utilization of specific AEF activities and the proportion of each?
3. Which components of AEF complexes (e.g. cyclic electron flow) are involved in catalytic activity and regulation?
4. How do subunits of individual AEF complexes interact; what controls these interactions?
5. What components of AEF pathways are post-translationally modified; how do such modifications impact protein structure, interactions, and activities?
6. Why are there two different NDH pathways (Type I and II); do differences in these pathways reflect their ability to use different substrates?
7. What are the functions of the chloroplast OMTs and TPTs in driving chloroplast-to-mitochondria electron flow and what regulates their activities?
8. How are the activities of transporters of phosphorylated sugars on the membranes of various cellular compartments integrated with chloroplast sugar-P transport?

With an initial plunge into the waters of AEF and associated metabolite trafficking, we are just beginning to realize the depth of those waters.

## Do flavodiiron proteins have multiple physiological roles in photosynthetic organisms?

### (Written by Yagut Allahverdiyeva, Henna Mustila, and Lauri Nikkanen)

Flavodiiron proteins (FLVs) play a crucial role in some photosynthetic organisms as an alternative electron transport pathway, allowing growth in fluctuating light intensities by providing a valve for the safe dissipation of excess electrons in the photosynthetic electron transport chain. FLVs drive a four-electron reaction reducing O_2_ to water downstream of PSI. This reaction is known as the Mehler-like reaction due to constituting a water–water cycle similarly to the Mehler reaction, or pseudocyclic electron transport (Fig. 7 #4, and see above section by Grossman). FLVs are found in various photosynthetic organisms ranging from cyanobacteria to vascular plants, but are absent in red algae and angiosperms. Cyanobacterial Flv1 and Flv3 have structural and functional similarities to FlvA and FlvB in algae and plants (Allahverdiyeva et al. 2015a, 2015b, Ilík et al. 2017). The reason for the loss of FLVs from angiosperms during evolution remains elusive. However, it may relate to increased efficiency of other electron sinks and protective mechanisms such nonphotochemical quenching, reactive oxygen species scavenging systems, and cyclic electron transport, which would have made retention of energy-wasting FLVs redundant or even disadvantageous (for discussion on this, see e.g. Alboresi et al. 2019b).

**Figure 8.**
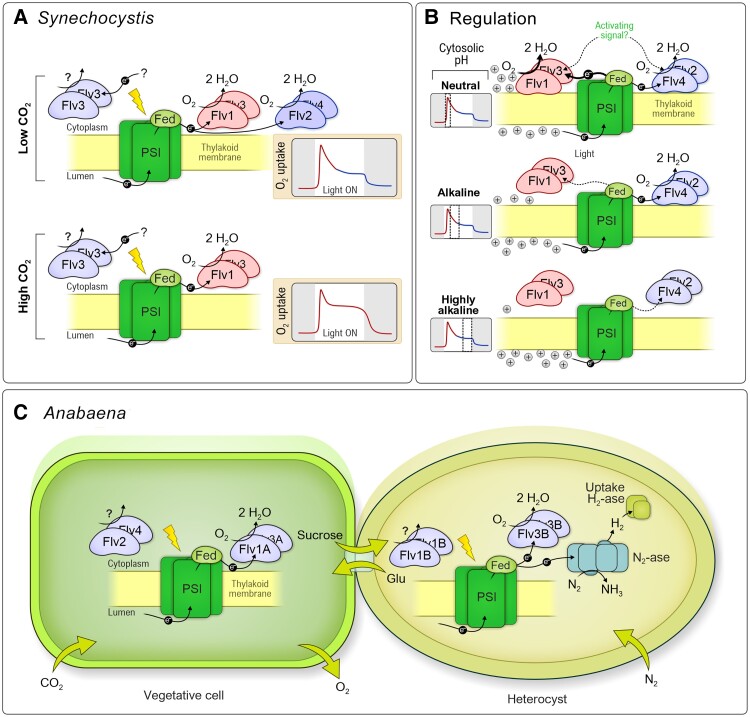
Function and regulation of FLVs in Synechocystis and Anabaena. **A)** In Synechocystis, Flv1/Flv3 and Flv2/Flv4 hetero-oligomers function in light-driven O_2_ photoreduction and their expression level is dependent on carbon availability. In addition, Flv3/Flv3 homo-oligomer catalyzes a distinct, unspecified reaction than the O_2_ photoreduction. **B)** The reversible association of different Flv1/3 and Flv2/4 hetero-oligomers with the thylakoid membrane of Synechocystis is controlled by the extent of the trans-thylakoid proton gradient. The + symbols refer to positively charged protons. See text for details. **C)** In filamentous Anabaena, Flv1A/Flv3A function in vegetative cells and Flv3B/Flv3B in heterocysts in O_2_ photoreduction, the latter configuration maintaining microoxic conditions to protect the N2-fixing enzyme nitrogenase in the light. The roles of Flv2/Flv4 and Flv1B/Flv1B in Anabaena are yet to be elucidated.

In the reference cyanobacterium Synechocystis (*Synechocystis* sp. PCC6803), the Flv1/Flv3 hetero-oligomer catalyzes strong but transient O_2_ photoreduction in the first minute of illumination or during increased light intensity under ambient CO_2_ (Santana-Sánchez et al. 2019), providing essential electron sink capacity during the initial phase of photosynthetic induction before full activation of the Calvin–Benson cycle (Fig. 8A). Accordingly, *flv* mutants (deficient in Flv1 or Flv3 or homologs FlvA and FlvB) demonstrate strong growth retardation under fluctuating light, where low background light is repeatedly interrupted by high-light pulses, causing strong acceptor-side limitation of PSI (Allahverdiyeva et al. 2013; Gerotto et al. 2016; Chaux et al. 2017; Jokel et al. 2018). Synechocystis Flv mutants show a less pronounced phenotype under milder fluctuating light conditions (higher background light) at pH 8.2, and no growth phenotype was observed at pH 7.5 (Mustila et al. 2016), suggesting an external pH-dependent effect on FLVs and growth that remains poorly understood.

In addition to Flv1 and Flv3, some *β*-cyanobacteria possess Flv2 and Flv4 proteins (Helman et al. 2003). These proteins also participate in Mehler-like reactions downstream of PSI, but with different kinetics, catalyzing O_2_ photoreduction that persists beyond the initial phases of photosynthetic induction (Santana-Sánchez et al. 2019; Fig. 8A). Unlike Flv1 and Flv3, Flv2 and Flv4 are not crucial during sudden increases in light intensity. In Synechocystis, Flv2 and Flv4 show strong downregulation under low light, high CO_2_ and high pH conditions (Eisenhut et al. 2012; Santana-Sánchez et al. 2019). Deletion of Flv2 and Flv4 alters the phenotype under high light, and overexpression of *flv4-2* operon provides resistance to high light (Bersanini et al. 2014). Notably, the presence of these proteins within an operon alongside Sll0218 suggests that some phenotypic changes could be attributed to the role of this small protein in PSII assembly and stabilization (Bersanini et al. 2017).

Heterocyst-forming N_2_-fixing cyanobacteria, such as Anabaena (*Anabaena* sp. PCC 7120), possess six FLVs (Fig. 8C). Flv1A and Flv3A are specific to vegetative cells and are crucial for survival under FL conditions. Interestingly, deletion of Flv3A affects N_2_ fixation and H_2_ metabolism in heterocysts, specialized N_2_ fixing cells, via downregulation of uptake hydrogenase, enabling H_2_ production even under oxic conditions (Santana-Sánchez et al. 2023). Investigations into the roles of Flv2 and Flv4 in Anabaena are yet to be conducted, although their expression pattern under different conditions suggests a function similar to that in Synechocystis. Flv1B and Flv3B are specific to heterocysts (Ermakova et al. 2013, 2014). Unlike other cyanobacterial FLVs, Flv3B can function as a homo-oligomer in the Mehler-like reaction, maintaining microoxic conditions under light. Importantly, while Flv1B is not involved in the Mehler-like reaction, its deletion strongly impacts N_2_ fixation, indicating a role in N_2_ metabolism (Ermakova et al. 2014).

### What are the functional oligomeric states of FLVs?

All FLVs contain an N-terminal metallo-β-lactamase-like domain harboring an O_2_ and/or NO reducing nonheme diiron center and a C-terminal flavodoxin-like (FMN) domain (Vicente et al. 2008). Oxygenic photosynthetic organisms contain an additional NAD(P)H:flavin reductase-like domain fused in the C-terminus. *In vitro* studies and modeling suggested that FLVs function in a head-to-tail homo-oligomeric configuration (Vicente et al. 2008) enabling inter-subunit electron transfer, between Fe–Fe of one monomer and FMN of the other. In Synechocystis, an in vivo hetero-oligomeric configuration (Flv1/3 and Flv2/4) is important for O_2_ photoreduction: Synechocystis strains lacking Flv1 but overexpressing Flv3 (thus Flv3/3 configuration) or lacking Flv3 but overexpressing Flv1 (Flv1/1) did not demonstrate O_2_ photoreduction (Mustila et al. 2016). However, having overexpressed homo-oligomers of Flv3 or Flv1 partly rescued the phenotype under FL, suggesting that homo-oligomers can function in unknown metabolic pathways and play a role in photoprotection that is independent of O_2_ photoreduction. Moreover, in Anabaena, the deletion of Flv1A did not result in total impairment of the Mehler-like reaction in air-level CO_2_ conditions where Flv2 and Flv4 are expressed (Santana-Sánchez et al. 2023). This suggests that Flv3A can catalyze O_2_ photoreduction in an Flv2/4-dependent manner either as a homo-oligomer interacting with Flv2/4 or by forming oligomers with Flv2 or Flv4.

### Can FLVs in photosynthetic organisms catalyze NO reduction?

FLVs can reduce NO into N_2_O in anaerobic bacteria (Vicente et al. 2008) and in *Chlamydomonas reinhardtii* (Burlacot et al. 2020). We cannot exclude a similar function in cyanobacteria, although the efficiency of this reaction as a sink is disputable. However, it may affect cell metabolism via signaling, and could be aligned with alternative electron donors. Recent data show that Synechocytsis Fed1, the most abundant of 11 ferredoxins in this cyanobacterium, can interact with and act as a donor to the Mehler-like reaction catalyzed by Flv1/3 and Flv2/4 (Nikkanen et al. 2020, 2023; Sétif et al. 2020). However, in vitro studies demonstrated that NAD(P)H is a donor for recombinant Flv1, Flv3, or Flv4 homo-oligomers (Vicente et al. 2008; Shimakawa et al. 2015; Brown et al. 2019), albeit with relatively low electron transfer rates. Interestingly, a protein–protein interaction was detected between Flv3 (but not Flv1) and the large isoform of FNR (FNR_L_) (Nikkanen et al. 2023). Flv3/FNR_L_ interactions could occur to facilitate electron transfer from NADPH to Flv3 homo-oligomers, functioning in a pathway to reduce an unknown acceptor, such as NO. Flv1-independent activity of Flv3 is also supported by 6-fold higher protein amount of Flv3 in comparison to Flv1 (Jackson et al. 2023). However, the physiological relevance of these interactions and Flv3-homo-oligomer-dependent electron transfer pathways remain unresolved.

### How is FLV activity regulated?

The Mehler-like reaction displays distinct kinetics where Flv1/3-dependent O_2_ photoreduction is transiently induced and then inactivated during the first minute of illumination. This strongly suggests that FLV activity is subject to post-translational regulation (Alboresi et al. 2019a). Recently, we proposed a model for pH-dependent regulation of FLV hetero-oligomer activity (Nikkanen et al. 2023), where alkalization of the cytosol during photosynthetic induction results in Flv1/3 hetero-oligomers being repelled from the thylakoid membrane due to their surface charge becoming more negative and their activity being thus decreased (Fig. 8B). Flv2/4 remains associated with the thylakoids longer after photosynthetic induction (i.e. in a more alkaline cytosol) due to large positively charged patches in Flv4. While this model explains many features of the observed kinetics of FLV activity, FLVs are likely subject to additional layers of post-translational regulation. The fact that we see a transient reduction of ferredoxin at all during dark-to-light transitions suggests that Flv1/3 hetero-oligomers are not yet fully primed in darkness, despite cytosolic pH being neutral and FLV hetero-oligomers being associated with the thylakoids. Therefore, it is likely that an additional unknown signal is required for the full light-dependent activation of Flv1/3 hetero-oligomers. Indeed, FLVs have been suggested to be redox-regulated via conserved cysteine residues, some of which in Flv1 and Flv3 are light-dependently reduced (Guo et al. 2014). It remains to be elucidated if thiol modulation or other post-translational modifications, such as phosphorylation, play a role in regulating FLV activity.

An additional question concerns how FLV activity is regulated in low versus high CO_2_ conditions. In Synechocystis, in high CO_2_, although Flv1 and Flv3 are downregulated, and Flv2 and Flv4 are not expressed (Santana-Sánchez et al. 2019), FLV-dependent O_2_ photoreduction activity is sustained at a higher level than in low CO_2_. This suggests a regulatory mechanism that connects FLV activity to carbon metabolism and possibly to carbon concentrating mechanisms.

## How is photosynthesis regulated during light fluctuations?

### (Written by Adrien Burlacot)

**Figure 9.**
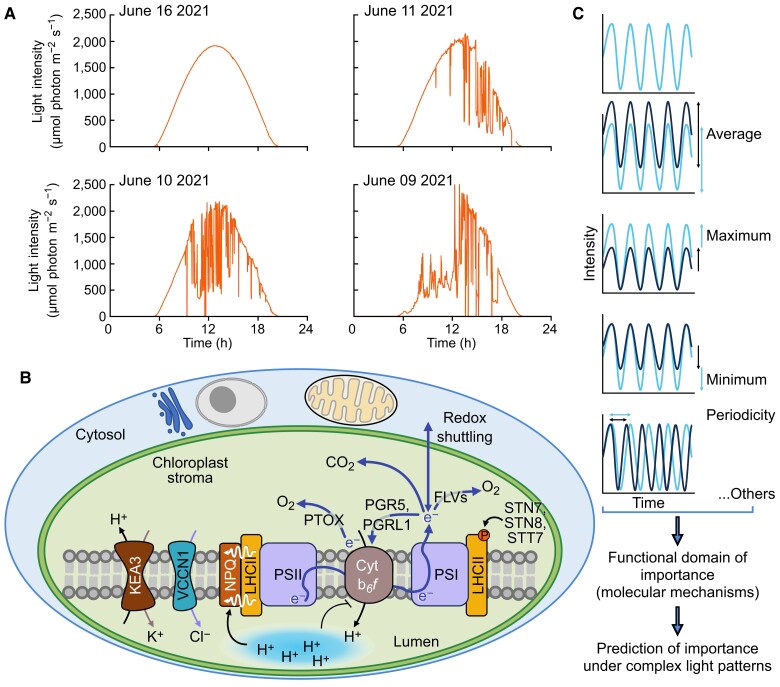
Acclimation mechanisms to light fluctuations. **A)** Light fluctuations generated by diurnal cycle and cloud coverage on top of a field canopy in June 2021. Data shown are light intensity of the photosynthetically active radiations throughout the time of the day from the Surface radiation Budget Network (SurfRAD, NOAA) at the Bondville Station, Illinois (40°N latitude; 88°W longitude). **B)** Main molecular mechanisms involved in tuning photosynthetic electron transport during artificial light fluctuations. The lumenal pH controlled by ion transport (mediated by KEA3, VCCN1, and the ATPase) and alternative electron transport (mediated by PGR5/L1 and FLVs), activates NPQ mechanisms and controls the photosynthetic electron flow at the cytochrome *b*_6_*f* (Cyt *b*_6_*f*) level. Phosphorylation of LHCII, which dynamically relocates LHCII to PSI, and redox exchange between the chloroplast and the rest of the cell are also involved in the dynamic regulation of photosynthetic electron transport. Shown in blue arrows are photosynthetic electron transport pathways. **C)** Example of characteristics of light fluctuations that can be explored to define the domain of importance of molecular mechanisms involved. The definition of such domains might help in predicting the set of mechanisms relevant to survive in a more complex light pattern.

Photochemical reactions happen extremely fast (on the picosecond timescale), but the redox reactions involved in the transport of electrons are orders of magnitude slower, and the metabolic reactions of carbon metabolism are even slower. This timescale discrepancy makes meeting metabolic energy requirements with light-energy-driven PS activity challenging to attain when light intensity is variable. In extreme cases of light fluctuation, photosynthetic electron generation can overflow electron transport or metabolic capacity, leading to PS degradation (Sejima et al. 2014) and potentially cell death. In many natural contexts, light rapidly fluctuates between a sub-saturating (low light, LL) to an over-saturating level (high light, HL) (Fig. 9A), and cells must cope with either excessive or insufficient amounts of energy and quickly switch between those states as light changes. The electron transport chain is a central hub of regulation in this context. Despite decades of investigations, how photosynthesis is dynamically controlled to provide chemical energy without becoming harmful during light fluctuations remains a thriving area of research (Morales and Kaiser 2020). This question has recently regained traction with groups showing that tuning the dynamics of light energy management can improve the productivity of photosynthetic organisms (Kromdijk et al. 2016; De Souza et al. 2022; Perin et al. 2023). Previous sections provided an overview of alternative electron flow pathways (Grossman) and the role of the pathway mediated by FLVs in some organisms (Allahverdiyeva et al.). Here, I discuss questions related to how organisms regulate the dynamics of which pathways and mechanisms are employed under fluctuating light conditions in different environments.

Many dynamic regulatory mechanisms of photosynthetic electron generation and management are triggered by low lumenal pH, which modulates electron transport at the level of the cytochrome *b*_6_*f* (Malone et al. 2021) and activates nonphotochemical quenching (NPQ) of excess absorbed light energy. Since the discovery of the role of thylakoid protein phosphorylation in response to 5 min LL/1 min HL treatment (Fig. 9B) (Tikkanen et al. 2010), similar artificial light patterns have been extensively used to identify proteins involved in the adjustment of the energetic status of the electron transport chain when light fluctuates. In model species of angiosperms, mosses, microalgae, and cyanobacteria (Fig. 9B) those include: (i) proton sensing proteins involved in NPQ which limit photochemistry and protect PS during HL (Cantrell and Peers 2017; Roach 2020; Steen et al. 2022; Niu et al. 2023); (ii) ion exchange across the thylakoid membrane mediated by VCCN1 and KEA3, which balances the proton motive force and controls NPQ and electron flow during the few minutes following a light change (Armbruster et al. 2016; Herdean et al. 2016), (iii) the plastid terminal oxidase (PTOX), which contributes to the redox poise of the thylakoid during the LL/dark phase of fluctuation (Nawrocki et al. 2019), (iv) cyclic electron flow controlled by PGR5 and PGRL1 proteins which tunes lumenal pH and electron flow during the first minutes of HL (Yamamoto et al. 2016; Yamamoto and Shikanai 2018; Storti et al. 2019), (v) FLVs, which dissipate the overflow of electrons and contribute to the low lumenal pH upon HL exposure within a few seconds by reducing O_2_ to water (Allahverdiyeva et al. 2013; Gerotto et al. 2016; Chaux et al. 2017; Jokel et al. 2018), and (vi) redox shuttles which rebalance the redox poise between the organelles (Yokochi et al. 2021).

Despite this extensive amount of knowledge, the complete set of proteins involved during light fluctuations is likely just beginning to be revealed. Indeed, acclimation mechanisms to HL or LL involve (i) posttranslational regulation of protein activity (Allorent et al. 2013) (on a timescale of milliseconds to tens of minutes), (ii) translation of mRNA (Durnford et al. 2003) (on a timescale of minutes), (iii) changes of mRNA levels (Mettler et al. 2014), (iv) structural changes of organellar membranes (Kirchhoff 2014b) or (v) leaf orientation or morphology (Feng et al. 2019) (on a timescale of minutes to hours/days). Hence, the preceding time in LL or in HL, the intensities of LL and HL, the shape of the light transient as well as how frequent light is shifting likely have a major impact on the identity and role of mechanisms involved in acclimation. The use of just one light fluctuation regime becomes thus limited both for discovering new proteins involved and for identifying domains where known proteins are important.

How do different light fluctuation characteristics change which mechanisms are involved in photosynthesis regulation? Reductionist approaches hold great promises in that regard. One of them, introduced by Nedbal and Březina (2002) and recently used on mutated strains by Shimakawa and Miyake (2018); Steen et al. (2022), and Niu et al. (2023) involves assessing the response of mutants impaired in photosynthetic regulatory processes to artificial periodic illumination (square-like or sine-like). Varying the periodicities used over short-term episodes of light fluctuations allows us to define the periodicity domain of photosynthetic regulatory processes, which are periodicity limits below or above which a regulatory mechanism cannot dynamically respond to a periodic fluctuation. Defining the periodicity domain of an acclimatory mechanism might allow predicting its importance during a more complex light fluctuation harboring a wide set of fluctuation periodicities. Note here that while this method is common in engineering sciences and relates to frequency-domain analysis, I refer here to the periodicity domain for notation simplicity. This method has allowed the unraveling of periodicity domains of proteins involved in NPQ: (i) in the green algae Chlamydomonas, NPQ magnitude and dynamics depend more on the light-harvesting complex stress-related 3 protein (LHCSR3) for a 1 min periodicity than for a 10 min periodicity (Steen et al. 2022), and (ii) in the angiosperm Arabidopsis, NPQ does not respond to dynamic light changes below a periodicity of 10 s while for periodicities of 10 to 60 s, the protein PsbS is critical for NPQ response; for longer periodicities, both PsbS and changes in the xanthophyll cycle contribute to the NPQ response (Niu et al. 2023). However, while defining periodicity limits of acclimatory processes can prove important, it remains limited to short-term episodes of light fluctuation and misses long-term acclimatory mechanisms. Another approach consists of growing/acclimating the photosynthetic organism under different patterns of light fluctuation and measuring how photosynthetic parameters are affected by the growth/acclimation conditions. Such an approach can reveal emergent phenomena in new mutants (Peng et al. 2020) and has recently revealed that (i) the average growth light intensity affects the quantum efficiency of PSII and the dynamics of ion transport across the thylakoids upon HL while, (ii) the variability of growth light changes the levels and de-epoxidation state of the xanthophyll-cycle pigments violaxanthin, antheraxanthin, and zeaxanthin (von Bismarck et al. 2023). Future work combining those two approaches with other key characteristics of light fluctuations will be important to understand how the variability of light influences the mechanisms involved in acclimation to light.

What happens in a natural light environment? While fluctuations of light intensity in nature can be complex (Fig. 9A), reductionist methods like the ones discussed above are powerful and should allow, when used systematically, the prediction of the identity and activity of acclimatory processes involved depending on key characteristics of light fluctuations (Fig. 9C). The periodicity of light fluctuation is a clear characteristic of natural relevance, as under a forest canopy, the movement of leaves forms gaps that let high-intensity sunlight pass typically during less than 1.6s (Pearcy 1990) while, at the top of the canopy, HL exposure typically lasts for a few minutes (Smith and Berry 2013). Such periodicity variability might even be larger in aquatic environments where rippling caustic effects of the water surface fluctuate light with periodicities of less than 100 ms (Schubert et al. 2001). While other light fluctuation characteristics influencing light acclimation mechanisms like average, maximal, and minimal light intensity have been explored to some extent (Allahverdiyeva et al. 2013; Jokel et al. 2018; von Bismarck et al. 2023); identifying a subset of key light characteristics that describes natural light fluctuations in different environment and quantifying their range remains to be done. An interesting possibility here is to apply signal decomposition methods currently used to study the output of photovoltaic panels in electrical engineering (Meyers and Boyd 2023) to define what are the most relevant light intensity characteristics present in different natural light environments.

Importantly, the presence or the absence of proteins regulating photosynthesis in different species can vary widely and this diversity and its implication are critical to study. For example, while FLVs are required for acclimation to 1min HL/5min LL fluctuations in many phototrophs, they are absent in angiosperms (Yamamoto et al. 2016), the latter most likely relying on other mechanisms to cope with sudden HL transients. The development of knowledge on the diversity of mechanisms across species (Sello et al. 2019), together with modeling the potential interaction of different mechanisms (Li et al. 2021a, 2021b), might allow molecular breeding of plants for adaptation to their local light environment in conditions where acclimation to light limits growth (Perin et al. 2019).

How is photosynthesis regulated during light fluctuations? The development of new tools (Cruz et al. 2016) and methods (Nedbal and Lazár 2021) to systematically look at different aspects of this question will bring new perspectives to the complex regulations of photosynthesis under light fluctuations. And the answers will most likely depend on which characteristics of light fluctuation are investigated.

## How can photorespiration impact stomatal opening?

### (Written by Michael Hodges)

Photosynthetic CO_2_ assimilation in embryophytes requires the uptake of atmospheric CO_2_ into the leaf. This is achieved by stomata, specialized epidermal structures composed of two guard cells. They possess partially overlapping signal transduction networks controlling stomatal pore size by modulating guard cell turgor pressure in response to abiotic and biotic cues including light, CO_2_, water potential, temperature, ozone, and pathogens. In this way, plants balance CO_2_ uptake and water loss with respect to fluctuating environmental conditions. Stomata are a target to be considered when designing future-proofed crops (Nguyen et al. 2023), therefore a complete understanding of how stomatal opening and closure are regulated is important. Although there is abundant knowledge concerning guard cell signaling networks and the H ^+^ -pumps, H ^+^ -coupled ion transporters, and ion channels involved in controlling stomatal movements (Saito and Uozumi 2019), how plant metabolism plays a role is less clear (Lemonnier and Lawson 2023). Mitochondrial activity (Nunes-Nesi et al. 2007) and cytosolic ATP (Lim et al. 2022) appear to be important for stomatal opening. Low blue light perceived by PHOT1/2 photoreceptors is involved in early morning guard cell starch degradation required for stomatal opening (Horrer et al. 2016). Photosynthesis has been shown to promote stomatal opening but its relative importance and cellular location (mesophyll cells versus guard cells) are still open to debate (Lemonnier and Lawson 2023). Recently, a role for photorespiration in stomatal functioning was suggested from experiments using photorespiratory mutants (Eisenhut et al. 2017; Flügel et al. 2017; Duminil 2019). If this is so, then how do photorespiratory cycle activity and/or photorespiratory-associated metabolites interfere with processes and signaling pathways that control stomatal opening/closure?

So what is photorespiration and which photorespiratory metabolites have the potential to influence stomatal opening/closure? Photorespiration is an essential metabolic process taking place in the light. It begins in chloroplasts with the oxygenase activity of Rubisco producing 2-phosphoglycolate (2-PG), which can inhibit the Calvin–Benson–Bassham cycle enzymes triose-phosphate isomerase and sedoheptulose 1,7-bisphosphate phosphatase as well as glycolytic phosphofructokinase (Flügel et al. 2017 and references therein). The photorespiratory cycle spans four cell compartments (chloroplasts, peroxisomes, mitochondria, cytosol) and requires eight enzymes to transform toxic 2-PG into useful 3-phosphoglycerate. To accomplish this, photorespiration leads to the production of H_2_O_2_ and NADH while liberating CO_2_ and ammonia during serine formation from glycine in mitochondria (reviewed in Eisenhut et al. 2019).

As early as the 1960s, short-term pharmacological studies using photorespiratory enzyme inhibitors showed a correlation between photorespiration and stomatal opening in the light. The addition of hydroxysulfonate (an inhibitor of glycolate oxidase, a peroxisomal enzyme producing H_2_O_2_ and glyoxylate from glycolate) to tobacco leaf disks (Zelitch and Walker 1964) and aminoacetonitrile (an inhibitor of glycine decarboxylase, a mitochondrial complex producing CO_2_) to rice leaves (Cho et al. 1987) provoked stomatal closure in the light. An association between photorespiration and stomatal opening/closure was further suggested because the inhibitory effect of aminoacetonitrile did not occur in detached rice leaves under low photorespiratory O_2_ air conditions and aminoacetonitrile application to maize (*Zea mays*) leaves (a C_4_ plant with low photorespiration) did not alter transpiration (Cho et al. 1987).

**Figure 10.**
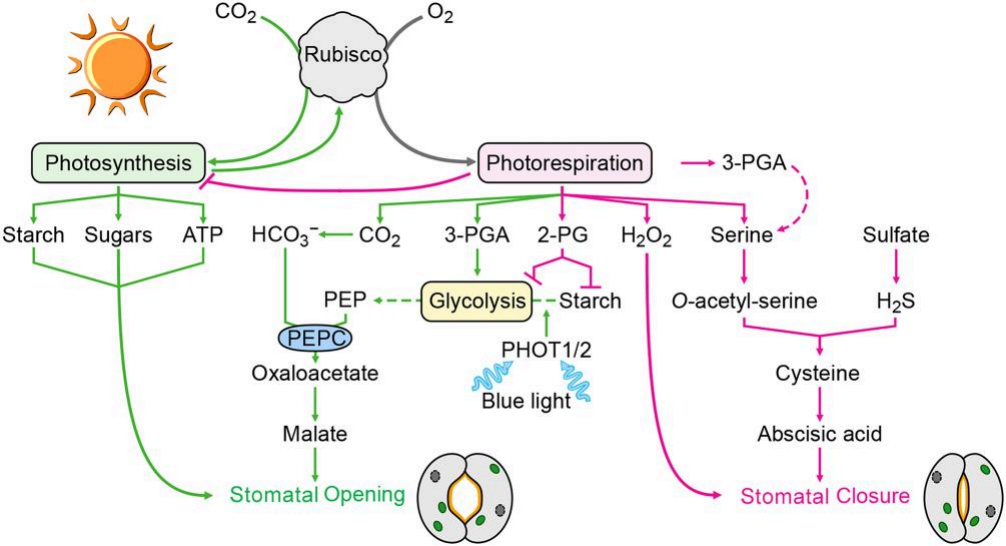
Potential connections between photorespiration and metabolic processes relevant to stomatal opening/closure. In the light, Rubisco is involved in both photosynthetic CO_2_ and photorespiratory O_2_ assimilation. Photorespiration produces a number of metabolites that have the potential to impact stomatal opening and closure. 2-PG can reduce starch biosynthesis and therefore blue light/PHOT-induced malate from starch degradation and thus limit early morning stomatal opening. 2-PG might also inhibit glycolysis and malate biosynthesis required for stomatal opening while photorespiratory CO_2_ and 3-phosphoglycerate (3-PGA) could be used to produce malate via PEP carboxylase (PEPC) activity for stomatal opening. Photorespiration also produces H_2_O_2_ and serine, and both could be linked to stomatal closure either as a signaling molecule or via the production of *O*-acetylserine required for sulfate-induced ABA biosynthesis, respectively. Photorespiration also negatively impacts photosynthetic activity thus reducing the capacity to produce starch, sugars, and ATP that are important for stomatal opening.

A renewed interest in photorespiration and stomata opening/closure has come from recent observations made using Arabidopsis photorespiration mutants (Fig. 10). Compared to wild-type (WT) plants, mutants lacking either phosphoglycolate phosphatase (*pglp1*), or serine hydroxymethyltransferase (*shm1*), or glycerate kinase (*glyk1*) showed higher and lower transpiration rates at high CO_2_ (10,000 ppm) and in ambient air, respectively (Eisenhut et al. 2017). This was later partially confirmed by direct stomatal aperture measurements using epidermal peels of *pglp1* leaves and light microscopy (Duminil 2019). Arabidopsis *PGLP1* antisense repression also led to lower stomatal conductance (*gs*) and transpiration rates when plants were transferred from high to ambient CO_2_ (Flügel et al. 2017). On the other hand, *PGLP1* overexpression gave higher *gs* values and transpiration rates in 40% O_2_ air; a high-photorespiration condition that increased leaf 2-PG amounts (Flügel et al. 2017). Lines overexpressing *PGLP1* also maintained high *gs* levels at an elevated temperature and after drought stress, conditions associated with increased photorespiration (Timm et al. 2019). Greenhouse-grown tobacco overexpressing glycine decarboxylase (GDC)-H subunit also exhibited higher *gs* although this was not observed in the field (López-Calcagno et al. 2019). By analyzing epidermal peels of complemented Arabidopsis *shm1* lines, SHM activity was found to impact salt- and ABA-induced stomatal closure (Liu et al. 2019).

So how can photorespiration impact stomatal opening/closing? There are a number of possible mechanisms that require investigation (Fig. 10).

- Transcriptional regulation could be involved since several photorespiratory mutants have deregulated genes involved in stomatal closure including *ABSCISIC ACID INSENSITIVE 1/2* (*ABI1*/2) (ABA biosynthesis), and *RESPIRATORY BURST OXIDASE HOMOLOGS D/F* (*RBOHD/F*) (reactive-oxygen production) and blue-light-induced stomatal opening (*PHOT1/2*) (Eisenhut et al. 2017). The signals and mechanisms involved are not yet known.
- Photorespiration could act by indirectly or directly impacting photosynthetic electron transfer and as a consequence ATP biosynthesis, which is required for stomatal opening. Photosynthetic processes have been shown to be affected by reduced photorespiratory cycle activity in mutants, including an attenuation of photosynthetic CO_2_ assimilation, modification of PSII redox potential, and an increase in energy dissipation mechanisms (Timm et al. 2012; Dellero et al. 2015, 2016; Messant et al. 2018). Indeed, a correlation between *gs* and photosynthesis exists (Nunes-Nesi et al. 2007; Lu et al. 2014).
- Changes in photorespiratory activity could also provoke metabolic regulation by interacting with and perhaps modulating metabolic pathways relevant to stomatal movements including the biosynthesis of starch, malate, and ABA.
- Photorespiration could impact blue-light-induced stomatal opening at the beginning of the day. It has been proposed that enzyme inhibition by 2-PG is a control loop adjusting stomatal conductance to photosynthesis-related C-ﬂuxes by altering C-allocation between RuBP regeneration and starch synthesis (Flügel et al. 2017). A correlation between PGLP activities, 2-PG levels, *gs*, and transpiration rates in PGLP transgenic lines (Eisenhut et al. 2017; Flügel et al. 2017; Timm et al. 2019) and reduced end-of-day leaf starch levels in photorespiratory mutants (Timm et al. 2012) support this hypothesis.
- Guard cell osmoregulation requires starch breakdown, triose-phosphate export from chloroplasts, and cytosolic PEP carboxylase (PEPC) activity for glycolytic malate production (Gehlen et al. 1996). Photorespiration produces 3-PGA and liberates CO_2_ and therefore this could fuel glycolysis and PEPC activity to promote guard cell malate biosynthesis and stomatal opening. On the other hand, a build-up of photorespiratory 2-PG might inhibit glycolysis and favor stomatal closure.
- Sulfate-induced stomatal closure upon drought is associated with guard cell-specific ABA biosynthesis (Batool et al. 2018). In photosynthetic tissues, photorespiration is the predominant serine biosynthesis route with 23% to 41% of photorespiratory serine leaving the cycle (Fu et al. 2023) to serve metabolic processes including sulfur-assimilation and cysteine biosynthesis (Abadie and Tcherkez 2019). In this context, photorespiratory serine could produce o-acetyl serine required for sulfate-associated cysteine biosynthesis and subsequent ABA production.

So is the photorespiratory cycle a good target to improve stomatal opening/closure traits in the field? Chloroplast photorespiratory by-pass plants with improved photosynthesis might be expected to impact stomatal opening. That said, no significant changes were found in Arabidopsis (Maier et al. 2012) or tobacco (South et al. 2019), and only rice showed a higher *gs* (Nayak et al. 2022). Not all photorespiratory mutants exhibit altered *gs* or transpiration when transferred from high CO_2_ to air (Dellero et al. 2015, 2016; Eisenhut et al. 2017) although antisense rice GOX showed a correlation between GOX activity and *gs* (Lu et al. 2014). Finally, reduction of end-of-day starch in *hpr1* leaves (Timm et al. 2012) was not associated with altered transpiration in the light (Eisenhut et al. 2017).

Many questions still remain unanswered such as how, where, and when does photorespiration impact stomatal opening/closing? It will be challenging but essential to answer these questions in the context of manipulating photorespiration to improve photosynthesis and plant yield since unwanted stomatal phenotypes could negatively impact water-use efficiency. Future directions to answer these questions should include determining stomatal kinetic responses to environmental cues in controlled atmospheric conditions differing in CO_2_/O_2_ levels to modulate photorespiration in attached leaves. Other important avenues of investigation include determining whether photorespiration in guard cells, mesophyll cells, or both cell types is important using cell-specific expression lines, and identifying how photorespiration impacts metabolites involved in stomatal movements using single-cell metabolomics associated with stable-isotope labeling.

## What adaptive changes make an enzyme suitable for the C_4_ carbon concentrating pathway, and how do critical and lineage-specific amino acid substitutions affect plant physiology?

### (Written by Clarisa E. Alvarez, Veronica G. Maurino, and Marcos A. Tronconi)

#### C_4_ proteins have accumulated different types of adaptive changes

**Figure 11.**
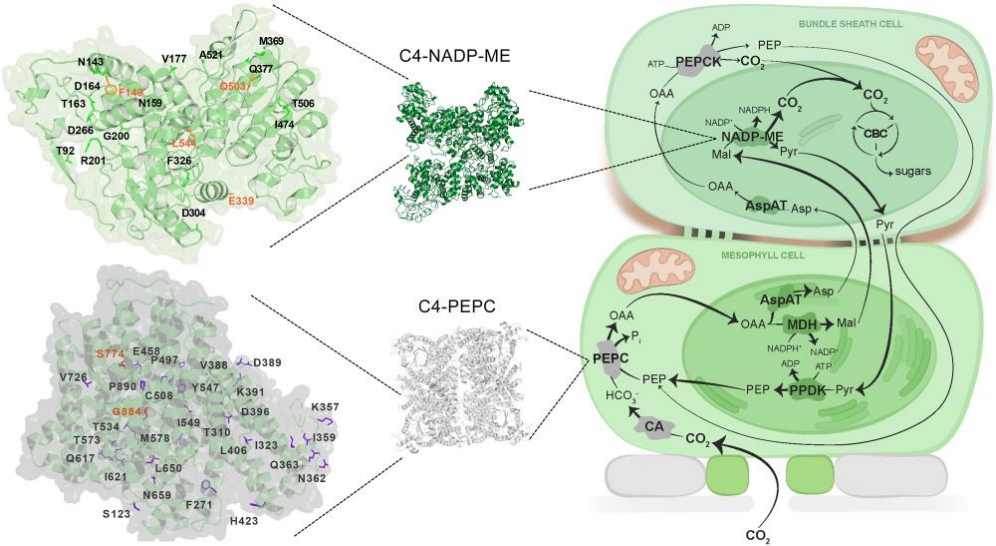
Aspects of the C_4_ pathway. On the right: a schematic representation of the C_4_ biochemical pathway as found in maize and *Flaveria*. The main fluxes are indicated by thicker arrows. CBC, Calvin–Benson–Bassham Cycle. In the middle: 3D structural representations of tetrameric C_4_-PEPC (bottom) and C_4_-NADP-ME (top). On the left: subunits of C_4_-PEPC and C_4_-NADP-ME, highlighting differentially substituted amino acids. Amino acids in orange represent critical substitutions. Amino acids in black represent lineage-specific substitutions. The protein structures for *Sorghum bicolor* C_4_-NADP-ME (PDB code 6C7N) and *Flaveria trinervia* C_4_-PEPC (PDB code 3ZGE) were modeled using PyMOL Molecular Graphics System, version 2.0 Schrödinger, LLC.

Photorespiration evolved as a selective response to Rubisco promiscuity, as a high-energy pathway to remove 2-phosphoglycolate, an unwanted reaction product. In C_3_ plants, photorespiration negatively affects fitness under conditions that reduce the relative concentration of CO_2_, such as high temperature, drought, and salinity. Some land plants have evolved a biochemical CO_2_ pump known as the C_4_ carbon concentrating pathway or C_4_ pathway. In the C_4_ pathway, CO_2_ is prefixed to form a C_4_ acid, which is then decarboxylated in the vicinity of Rubisco. These reactions are usually spatially separated between two cell types (Fig. 11A) (Maier et al. 2011; Drincovich et al. 2016). By increasing the CO_2_ concentration around Rubisco, C_4_ plants gain a beneficial reduction in photorespiratory flux. The evolution of the C_4_ pathway resulted in the coordinated acquisition of new anatomical and biochemical features (Sage and Zhu 2011; Alvarez and Maurino 2023). It is widely accepted that anatomical C_4_ traits were acquired prior to C_4_ biochemistry (Christin and Osborne 2014). On this basis, C_4_ pathway genes were formed by co-opting copies of duplicated genes that already existed in C_3_ species (Christin et al. 2013). In most cases, the same gene lineage has been selected repeatedly by independent C_4_ origins. The bias towards co-opted genes may be related to fewer key genetic changes in their regulatory and coding regions, which need to accumulate rapidly to acquire the C_4_ function (Copley 2020).

Some of the adaptive amino acid changes are critical for C_4_ function: if one of these is mutated back to the amino acid present in the C_3_ version of the enzyme, there is a loss of crucial C_4_ biochemical properties (kinetic, regulatory, and/or structural). In addition, other adaptive changes have occurred to optimize the C_4_ function, either (i) by counteracting perturbations in protein properties caused by previous critical C_4_ changes, such as a reduction in stability of the protein structure, or (ii) by adapting the enzyme activity to the new physiological environment that accompanies C_4_ activities. Candidates for critical adaptive amino acid changes can be identified through an analysis of protein sequences from different C_4_ lineages in the form of strictly differentially substituted positions (Alvarez et al. 2019; Hüdig et al. 2022). A strictly differentially substituted position is one where the protein sequences in the C_4_ species contain an identical amino acid, while a different amino acid is shared by all C_3_ sequences. The data obtained so far indicate that critical adaptive changes for C_4_ innovations have evolved with a high degree of convergence between distant C_4_ lineages, as in the case of PEPC (Christin et al. 2007; Rosnow et al. 2014; Lyu et al. 2021), or restricted to genetically close C_4_ lineages, as in the case of NADP-ME (Alvarez et al. 2019; Alvarez and Maurino 2023) and NAD-ME (Tronconi et al. 2020; Hüdig et al. 2022), demonstrating that alternative solutions to critical C_4_ requirements exist. Such sequence analyses also reveal a large number of molecular adaptations that are lineage-specific (Fig. 11, B and C).

### Innovation is easy, optimization is complicated: how can we analyze the impact of adaptive changes of C_4_ proteins?

C_4_ innovations can be achieved by a rather small number of critical changes, some of which evolved divergently while others appear to have occurred convergently across C_4_ lineages. In this regard, when comparing the differentially substituted residues between the C_4_-PEPC from Poaceae, Chenopodiaceae, and Asteraceae, which are genetically very distant, only one residue underwent an identical substitution in all three lineages. This substitution causes the C_4_-PEPC to acquire affinity values for substrates similar to those of non-C_4_ isoforms. This suggests that only minimal changes to the C_3_ ortholog may be required to perform the specialized C_4_ function. However, the presence of a large number of lineage-specific amino acid substitutions in C_4_ proteins indicates that the optimization of C_4_ function is much more complicated and may depend on lineage-specific physiological details. During the evolution of C_4_ biochemistry, the specific compartmentalization of enzyme activities and increased protein abundances created a new cellular environment (Lyu et al. 2021). In addition, the evolution of different biochemical and anatomical C_4_ subtypes imposed different physiological constraints between the C_4_ lineages. As a result, C_4_ enzymes coordinately adapted to the new metabolic context and the need for high carbon flux by fine-tuning their function through lineage-specific amino acid substitutions. An illustration of this process was provided by Lyu et al. (2021), who found that several genes encoding proteins associated with the C_4_ pathway in *Flaveria* species showed highly coordinated patterns of gene expression and modification in protein sequence.

The details of the molecular evolution of most C_4_ enzymes are still largely unknown. Present knowledge is based on the analysis of C_4_ enzymes from a small number of species in a few C_4_ lineages. Thus, it is not yet clear whether critical C_4_ changes are generally convergent, or whether they depend on the co-opted gene or on specific features of C_4_ subtypes. So, what adaptive amino acid substitutions make a protein suitable for C_4_ biochemistry in each lineage? To answer this question, much more data from more species is needed to allow intra- and inter-lineage analyses to associate critical C_4_ changes with specific amino acid replacements.

Data on the kinetic and regulatory properties of an enzyme are rarely collected under conditions that mimic physiological ones, and they are therefore not sufficient to fully understand an enzyme's contribution to fitness. In fact, the performance of an enzyme depends on the physiological environment, emphasizing the importance of studying how adaptive changes affect plant physiology. At present, our knowledge of the in planta effects of adaptive changes is at a nascent stage. For example, in maize, *Amaranthus*, and *Flaveria*, the high activity level and low malate sensitivity of C_4_-PEPC were attributed to the phosphorylation state of the enzyme (Jiao and Chollet 1988; Tsuchida et al. 2001; Avasthi et al. 2011). However, the lack of phosphorylation of C_4_-PEPC from *Flaveria bidentis* was not crucial for PEPC activity or high photosynthesis rates under standard greenhouse conditions (Furumoto et al. 2007). Another example is the pH-dependent inhibition by malate of C_4_-NADP-ME from maize, sorghum (*Sorghum bicolor*), and *Setaria italica* (Alvarez et al. 2019). The question is, how does this regulatory property—exclusive to the C_4_ isoform—affect plant physiology in a day-night cycle?

Several genetic engineering strategies to introduce a “C_4_-like pathway” into C_3_ plants have failed to improve photosynthesis, most likely due to the lack of adaptation of the C_4_ enzyme to the intracellular C_3_ environment. There is a need to assess the biochemical and molecular characteristics of C_4_ enzymes in planta to understand how and under what conditions specific adaptive changes may affect plant physiology. In the future, new technologies such as CRISPR will facilitate the introduction of wild-type and mutated versions of C_4_ enzymes in C_3_ and C_4_ plants and the further analysis of their impact on plant metabolism and physiology. For example, to assess whether lineage-specific adaptive changes are related to the optimization of C_4_ function, C_4_ mutant plants lacking their own photosynthetic C_4_-enzyme but expressing the C_4_ isoform of a distant lineage could be generated (e.g. C_4_-PEPC from grasses expressed in the C_4_*Flaveria trinervia* C_4_*-pepc^−^* mutant). In addition, C_4_ mutant plants expressing a distant C_4_ enzyme that contains amino acid substitutions specific to the lineage in which it is expressed could also be tested. These analyses would contribute to our overall understanding of the regulatory processes that are involved in the intricate dynamics of C_4_ plant metabolism.

## Conclusions

The questions covered here span a wide range of processes related to oxygenic photosynthesis. A few common themes and conclusions can nonetheless be drawn from these diverse topics. First, protein interactions are critical and govern almost all cellular processes. The most exciting and important answers to unsolved questions will come from identifying the key proteins and their interacting partners, where and when the interactions take place, how they interact, and how they are regulated. A key aspect of many of the topics covered here is an emphasis on the importance of timing; when are molecular mechanisms active or interacting together? This is likely true for any biological system; therefore, answers to these questions will provide fundamental insights not only into photosynthesis but many other biochemical processes as well. Second, the core photosystems PSI and PSII are highly conserved among photosynthetic organisms, but many aspects of their regulation, from light harvesting to how the resulting chemical energy is dispersed and used in the cell and how excess energy is dissipated, are highly diversified across the plant kingdom. Therefore, more work is needed not only on the historical model systems (e.g. Chlamydomonas, Arabidopsis) but on developing new model systems and understanding the diversity of photosynthesis in all its forms. Our discussions of the above questions further suggest that we are entering an era of understanding—and being able to interrogate—the ecophysiology of photosynthesis at the molecular level, which will be critical to understanding the diversity of photosynthetic regulatory mechanisms.
